# A systematic review on visual scanning behaviour in hemianopia considering task specificity, performance improvement, spontaneous and training-induced adaptations

**DOI:** 10.1080/09638288.2023.2243590

**Published:** 2023-08-10

**Authors:** Eva M. J. L. Postuma, Joost Heutink, Sarah Tol, Josephien L. Jansen, Jan Koopman, Frans W. Cornelissen, Gera A. de Haan

**Affiliations:** a Department Clinical and Developmental Neuropsychology, Faculty of Behavioral and Social Sciences, Rijksuniversiteit Groningen, Groningen, The Netherlands; b Royal Dutch Visio, Centre of Expertise for Blind and Partially Sighted People, Huizen, The Netherlands; c Laboratory for Experimental Ophthalmology, University Medical Center Groningen, University of Groningen, Groningen, The Netherlands

**Keywords:** Hemianopia, scanning, eye movements, mobility, reading, searching

## Abstract

**Purpose:**

People with homonymous hemianopia (HH) benefit from applying compensatory scanning behaviour that limits the consequences of HH in a specific task. The aim of the study is to (i) review the current literature on task-specific scanning behaviour that improves performance and (ii) identify differences between this performance-enhancing scanning behaviour and scanning behaviour that is spontaneously adopted or acquired through training.

**Materials and methods:**

The databases PsycInfo, Medline, and Web of Science were searched for articles on scanning behaviour in people with HH.

**Results:**

The final sample contained 60 articles, reporting on three main tasks, i.e., search (*N* = 17), reading (*N* = 16) and mobility (*N* = 14), and other tasks (*N* = 18). Five articles reported on two different tasks. Specific scanning behaviour related to task performance in search, reading, and mobility tasks. In search and reading tasks, spontaneous adaptations differed from this performance-enhancing scanning behaviour. Training could induce adaptations in scanning behaviour, enhancing performance in these two tasks. For mobility tasks, limited to no information was found on spontaneous and training-induced adaptations to scanning behaviour.

**Conclusions:**

Performance-enhancing scanning behaviour is mainly task-specific. Spontaneous development of such scanning behaviour is rare. Luckily, current compensatory scanning training programs can induce such scanning behaviour, which confirms that providing scanning training is important.

**IMPLICATIONS FOR REHABILITATION**
- Scanning behaviour that improves performance in people with homonymous hemianopia (HH) is task-specific.
- Most people with HH do not spontaneously adopt scanning behaviour that improves performance.
- Compensatory scanning training can induce performance-enhancing scanning behaviour.

## Introduction

Timely detection and recognition of relevant visual information is critical when performing various daily activities. Yet, when someone is deprived of perception in part of their visual field, obtaining this visual information becomes more challenging [1,2]. People with homonymous hemianopia (HH; i.e., blindness of either the left or right half of their visual field) encounter such challenges daily, often leading to considerable difficulties with various activities. Amongst others, they report difficulties with mobility, search, and reading activities [3–6]. For example, obtaining a complete overview of their environment while walking is challenging, and failing to do this adequately may even result in bumping into objects or other people [2,7,8]. Such difficulties negatively affect participation, independence, and quality of life [4,5,9,10]. For this reason, people with HH should be provided with optimal rehabilitation programs to overcome these difficulties.

People with HH may be able to reduce difficulties with these activities by adjusting their eye and head movements in order to effectively compensate for the visual field loss. In current rehabilitation practice, people with HH are trained to use such compensatory scanning to improve performance in, amongst others, search, reading, and mobility activities [8,11–16]. These current compensatory scanning training programs have been shown to increase performance on search, reading, or mobility tasks. Note that in this review, the term scanning is used in the broad sense of the word, referring to all types of viewing behaviour made during such activities, and tasks refer to experimental tasks designed to investigate the performance of an activity (see definition list in Supplementary Appendix D). While the results of compensatory scanning training programs appear to be promising, it is less clear which specific training-induced adaptations to scanning behaviour contribute to the improvements in specific tasks. Moreover, it remains a question whether the trained scanning behaviour is also the behaviour that improves task performance.

Even without having received training, people with HH may still spontaneously adapt their visual scanning behaviour [17,18]. For example, some people with HH make more eye movements towards their blind hemispace compared to their visible hemispace [17]. Moreover, their scanpaths are longer than in people with unimpaired vision. Whether such spontaneous adaptations improve or reduce task performance also remains a question. This lack of knowledge on how spontaneous adaptations relate to performance-enhancing scanning behaviour (i.e., scanning behaviour that was found to be positively related to task performance) limits the development and improvement of compensatory scanning training because it is still debatable whether people with HH should or should not change these spontaneous adaptations towards another, possibly more effective scanning behaviour.

Another challenge for compensatory scanning training is the apparent task specificity. Compensatory scanning training that practises mobility did show improvements in a mobility obstacle avoidance task but did not improve performance on reading and search tasks [11,12]. Similarly, improvements in search tasks did not transfer to reading tasks [14,19] and vice versa [19]. These findings may indicate that performance-enhancing scanning behaviour is task-specific. Moreover, this task-specificity should thus be incorporated in compensatory scanning training.

Hence, people with HH may not receive optimal compensatory scanning training for a particular task, because the specifics of the scanning behaviour that improves performance for that particular task are yet unknown. It cannot be ruled out that people with HH may even learn scanning behaviour that in fact reduces task performance. Therefore, it is of major importance to identify performance-enhancing scanning behaviour and its task-specificity. An earlier systematic review took the first step towards identifying performance-enhancing scanning behaviour in people with HH by providing an overview of spontaneous adaptations to scanning behaviour in exploration tasks [17]. It was found that generally people with real HH differ in scanning behaviour from people with unimpaired vision and simulated HH, indicating that people with HH spontaneously adapt their scanning behaviour due to their visual field defect. It also implies that short-term adaptations may differ from long-term adaptations in scanning behaviour or that these adaptations may be related to other impairments resulting from the acquired brain injury. Lastly, the systematic review showed that compensatory scanning training can modify scanning behaviour. While this review shows interesting insight into scanning behaviour in HH, it is still unclear whether these general spontaneous or training-induced adaptations in scanning behaviour relate to scanning behaviour that improves performance in specific tasks.

Therefore, the next step to take is to evaluate the task-specificity of performance-enhancing scanning behaviour in people with HH. Moreover, it is critical to understand how performance-enhancing scanning behaviour relates to both spontaneous and training-induced adaptations in scanning behaviour. The present systematic review takes this step. It aims to serve as a guide for future research into scanning behaviour in people with HH and contributes to knowledge on whether and how scanning training should be tailored to optimise performance on specific tasks.

## Method

### Literature search

The databases PsycInfo, Medline, and Web of Science were searched for journal publications on scanning behaviour in people with HH. Articles up to 15 November 2021 were considered. The search strategy is presented in Box 1. Terms in PsycInfo and Medline were searched in all fields. Terms in the Web of Science were searched by topic.

Box 1 Search strategies

(Hemianopia OR hemianopic OR hemianopsia OR hemiopia OR homonymous field defect* OR cerebral blindness OR cortical blindness OR visual field defect* OR visual field disorder*)

AND

(Scanning performance OR scanning behaviour OR scanning behaviour OR saccadic behaviour OR saccadic behaviour OR saccadic adaptation* OR saccades OR visual scanning OR visual performance OR visual search OR search task OR visual exploration OR oculomotor compensation* OR oculomotor pattern* OR oculomotor response Or oculomotor adaptation* OR ocular fixation OR fixation performance OR gaze OR gazing OR viewing behaviour OR viewing behaviour OR viewing efficiency OR eye-fixation OR eye fixation OR visual fixation OR eye-tracking OR eye tracking OR eye movement* OR eye–movement* OR EOG OR electrooculography OR electro oculography OR scanpath OR scanpath OR head movement* OR head-movement* OR eye-head coordination)

### Eligibility criteria

Articles were included when they met the following criteria: (a) the study included human adults with homonymous hemianopia (b) the study objectively measured scanning behaviour by using an eye tracker and/or head motion tracker system. Articles were excluded when they were: (a) published in another language than English, Dutch, or German, (b) not peer-reviewed (e.g., supplements, meeting abstracts, notes, letters to editors), (c) reviews, (d) not explicitly reporting scanning behaviour (i.e., actively exploring the environment for salient task-related information by using head and/or eye movements) as an outcome variable, (e) about transient field defects, (f) using eye-tracking only for diagnostic or perimetric assessment, (g) describing an HH participant group that included people with additional visual or attentional discords, such as neglect, (h) case studies. The reference list of reviews was checked for additional articles that fulfilled the eligibility criteria but were not found by the literature search. Lastly, the reference lists of eligible articles were searched in order to identify additional publications that were not identified during the database search.

### Protocol

The literature search was performed according to the guidelines of Preferred Reporting Items for Systematic Reviews and Meta-Analyses (PRISMA, 2009). Researcher EP performed the inclusion and exclusion of the articles. All excluded articles were additionally checked for inclusion and exclusion by researcher ST to make sure no relevant articles were missed. The records after the removal of duplicates were first screened by title and abstract. Full texts of the remaining articles were then assessed on their content for eligibility.

### Data extraction

From all included articles, data were extracted on participant characteristics, tasks in which scanning behaviour was examined, task performance, training, and scanning behaviour. In this review, performance-enhancing scanning behaviour refers to scanning behaviour that was found to be positively related to performance on a certain task. Performance-enhancing scanning behaviour in people with hemianopia was examined by extracting data on statistical group comparisons between high-performing people with HH and low-performing people with HH. Outcomes on scanning parameters that were linked to task performance by correlation or regression analysis with the scanning parameters as dependent variables were extracted as well. Spontaneous adaptations in people with HH were assessed by extracting results on statistical group comparisons between people with HH and unimpaired vision. To determine the training-induced adaptations in scanning behaviour in people with HH, data were extracted from the articles that describe compensatory scanning training. Compensatory scanning training was defined as one or more sessions in which people with HH had to perform exercises with the learning goal of applying more efficient scanning behaviour. The training could consist of performing exercises related to daily-life activities, such as mobility, reading, or searching, or exercises in which a specific scanning behaviour is practised, such as making longer saccades or a systematic scanning pattern. Articles were included when scanning behaviour before and after an intervention was measured using an exercise that was not identical to the training exercises. Outcomes on statistical pre- and post-analyses of scanning behaviour were extracted from the articles.

When comparing data on scanning behaviour between articles, the reference system needs to be considered [20]. Remote eye trackers (i.e., not attached to the head, but placed in a fixed position at a short distance from the person) are often used in laboratory studies and record eye movements in a gaze-in-world reference system (i.e., the coordinates refer to positions in the world). Mobile eye trackers (i.e., attached to glasses that the person is wearing) are often used in real-world studies and obtain eye movements in an eye-in-head reference system (i.e. the coordinates refer to positions relative to the viewing perspective of the head/head-mounted eye tracker). The same movement may result in different outputs when measured with a remote or mobile eye tracker. For example, when fixating one’s eyes on a fixed position in space, while rotating one’s head, the output of the remote eye tracker does not indicate a change (no eye movement detected), while the output of the mobile eye tracker indicates that an eye movement is being made. Therefore, both the reference system and the movement restriction of the head (free or restricted) were extracted from the articles in order to allow correct interpretation of the results.

Scanning behaviour can be analysed by means of event classification (e.g., categorising data into fixations, saccades, or head movements and shifts between the left and right hemispaces) or by analysing the sample-by-sample eye-tracking position data without event classification. In this review, scanning parameters that were obtained from analysing sample-by-sample position data without event classification is referred to as glances.

Scanning parameters related to the lateralization (i.e., left vs. right or blind vs. seeing side) of scanning behaviour can be analysed by two distinct methods. First, scanning parameters can be related to scanning the left or right half of a stimulus, such as a picture. This method was called hemispace analysis. The scanning parameters were described as “in blind hemispace” (i.e., the side of the stimulus corresponding to the blind side of the visual field) or “in visible hemispace” (i.e., the side of the stimulus corresponding to the visible side of the visual field). Second, scanning parameters can be described in terms of direction. Saccades towards the unaffected or affected side (regardless of the position of the start and end of the saccade) were described as “directed towards.” This analysis method was called the directional analysis.

### Data categorization

After data extraction, the articles were first categorised by the identified tasks in which scanning behaviour was measured. Within these task categories, the articles were checked for reporting on performance-enhancing scanning behaviour, spontaneous adaptations in scanning behaviour, or training-induced adaptations in scanning behaviour. In addition, the data on scanning behaviour was organised by main characteristics of scanning behaviour, to be able to compare the different scanning behaviour parameters described in the literature.

### Quality assessment

A quality assessment was performed on the articles that included data on performance-enhancing scanning behaviour, spontaneous adaptations in scanning behaviour, or training-induced adaptations in scanning behaviour in task categories that included more than 10 articles. The Joanna Briggs Institute case-control studies appraisal tool was used for articles describing performance-enhancing scanning behaviour and spontaneous adaptations in scanning behaviour [21]. This appraisal tool contains 10 assessment criteria. For the articles describing training-induced adaptations in scanning behaviour, the Joanna Briggs Institute quasi-experimental appraisal tool was used containing nine assessment criteria [22]. For both appraisal tools, each criterion was rated with a “yes,” “no,” “unclear,” or “not applicable.” Criteria rated with “yes” were given one point of which the total score was calculated. A total score below six was defined as a low-quality score. Authors EP and ST performed the quality assessment independently. Cohen’s kappa was calculated from the number of agreements on each criterion of this independent quality assessment. Afterwards, the authors reached an agreement on the final quality of each article based on the criteria.

## Results

**Figure 1.**
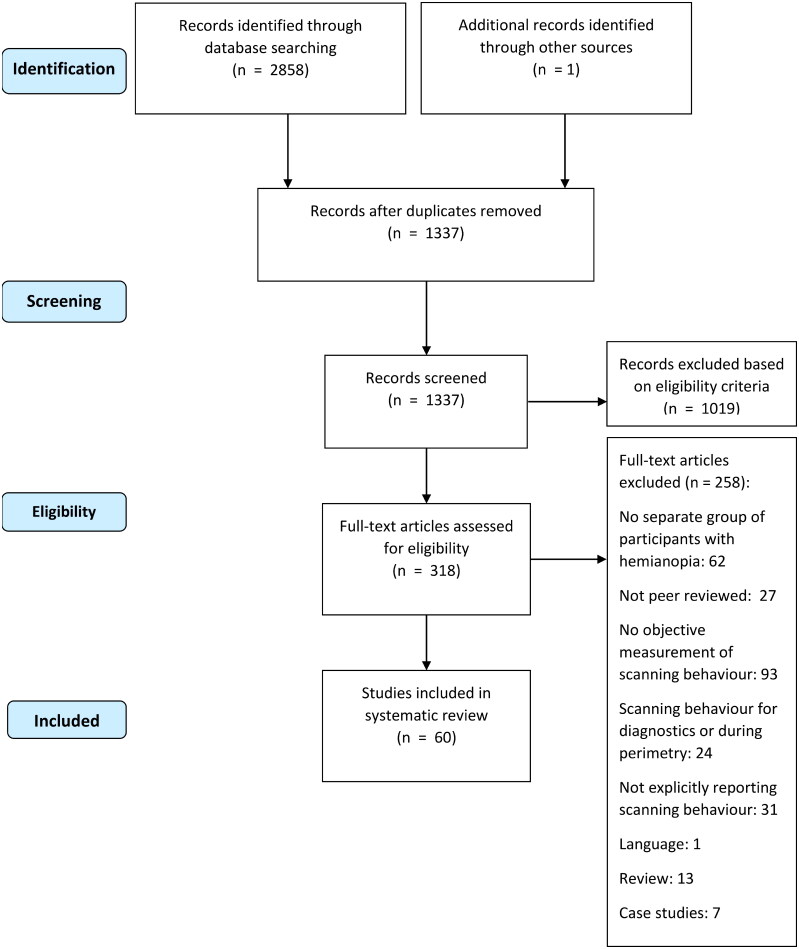
Flow diagram of article search and selection.

The search yielded a total of 2858 results (Figure 1). After the removal of duplicates and including one article from the references of reviews, 1337 records remained. Of these, 1019 were rejected based on title and abstract. Full-text eligibility assessment was performed on the remaining 318 articles which resulted in a motivated exclusion of another 258 articles. The final sample of eligible articles contained 60 articles. This final sample was found to describe eight task categories in which scanning behaviour was examined. Table 1 includes a description of the task categories, as well as a summary of the type of scanning behaviour examined in the articles.

**Table 1.** Allocation of articles to task categories with an overview of type of scanning behaviour studied.

Task	Definition	Total	PESB	SASB	TASB	References	Tables^a^
Search	Tasks in which participants have to find specific features or objects	17	2	9	6	[2,8,13,18,23–35]	T 2–5
Reading	Tasks in which participants have to read a text or several words	16	1	6	5	[13,24,29,31,35–46]	T 6–9
Mobility	Tasks in which subjects have to move through an environment while detecting task-relevant visual information	14	6	3	0	[27,47–59]	T 10–12
Imagery	Tasks in which a participant had to imagine specific images or recall previously viewed images.	7	0	3	0	[60–66]	S 1–2
Natural viewing	viewing a scene without any specific instruction	5	0	3	0	[67–71]	S 3–4
Line bisection	A version of the line bisection task was performed	4	0	2	0	[72–75]	S 5–6
Creating	Tasks in which participants have to create an object by for example drawing or building	2	0	1	0	[69,76]	S 7–8

PESB: performance-enhancing scanning behaviour; SASB: spontaneous adaptations in scanning behaviour; TASB: training-induced adaptations in scanning behaviour. Some articles describe several categories of scanning behaviour.

^a^Tables of searching, reading, and mobility are presented in this article.

Tables of natural viewing, imagery, following targets, line bisection, and creating are presented in the Supplementary Material Appendix A.

NB: Five articles were grouped under two categories, since they reported on two different tasks, such as a search and a reading task [13,23,27,29,35].

We will present and discuss the results of scanning behaviour for the three most studied categories; search, reading, and mobility tasks. The results for the remaining five identified categories can be found in the Supplementary Appendix A. Scanning behaviour as described in the articles was categorised by the number of scanning events (e.g., number of fixations, saccades, head movements), repetitions of scans (e.g., number of repetitions of fixations or repetitions of scanpath), duration of scanning events (e.g., fixation duration, saccadic duration), length of scans (e.g., total scanpath length, saccadic, or head movement amplitude), area distribution of scanning (e.g., proportion of fixations in the blind hemispace, proportion of fixations in the central area), dispersion of scanning (e.g., variance in fixation or glance positions), and scanning span (e.g., mean glance depth into the blind hemispace from midline). No article reported on every scanning parameter.

### Quality assessment

Five articles scored below six on the critical appraisal tool, indicating a low quality [8,28,31,36,37]. These five articles consisted of one article reporting on performance-enhancing scanning behaviour in search tasks [8], one article reporting on spontaneous adaptations in scanning behaviour in search tasks [28], and three articles reporting on spontaneous adaptations in scanning behaviour in reading tasks [31,36,37]. All other articles scored between six and eight. For more information on the quality of each article, see Supplementary Appendix B. The total Cohen’s kappa agreement before reaching consensus was 0.73. In the Supplementary Appendix C, Cohen’s kappa agreements for interrater reliability are shown separately for performance-enhancing scanning behaviour, spontaneous adaptations, and training-induced adaptations in scanning behaviour for the main task categories.

### Search tasks

The data from the articles that describe scanning behaviour of people with HH during search tasks are presented in Tables 2–5. Further clarification on the terms performance-enhancing scanning behaviour, spontaneous adaptations in scanning behaviour, and training-induced adaptations in scanning behaviour is provided in a definition list (Supplementary Material Appendix D).

**Table 2.** Descriptives of articles reporting scanning behaviour during search.

Article	*N*	Gender (%M)	Age (M (SD))	Right-sided HH	Left-sided HH	Quadrant-anopia^a^	Time since onset (range)	Task	Group division based on performance or used performance measure for correlation/regression analysis	Training
[2]	HH = 29	79	nm	15	14	0	32 Months (6–157)^b^	Counting all the dots that were presented on a screen in irregular patterns		
	UV = 16	44	40 (16–71)^b^							
[8]	HPH = 14	nm	19–76	30	30	0	1.5–12 Months	Counting all the dots that were presented on a screen in irregular patterns	Subjects with hemianopia with search times within the range of subjects without hemianopia were placed in the HP group. All other subjects with hemianopia were included in the LP group.	
	LPH = 36		LPH = 36							
	HH = 14	64	44 (23)	8	6		11 Weeks (6–18)^b^	Counting all the dots that were presented on a screen in irregular patterns		Visual scanning training with search exercises. The training consisted of two phases. In the first phase, participants were trained to make large saccadic eye movements towards the blind hemispace. In the second phase 5–30 different shapes or letters were presented. Participants searched for all targets with a specific feature (between 5 and 8).
[13]	HH = 20	85	59 (12)	6	6	RT = 3, RB = 1	28.9 (28.4)	Counting all the dots that were presented on a screen in irregular patterns		Visual scanning training with reading exercises. Text: 300 single words (3 to 13 letters, 10 units of 30 words, 45 min total) were read during one session. Participants were instructed to perceive each word as a whole before reading it aloud by shifting their gaze from the centre to the blind hemispace. An average of 11 sessions within 2 weeks.
	HH = 20	85	59 (14)	6	6	RT = 3, RB = 1	31.0 (47.0)	Counting all the dots that were presented on a screen in irregular patterns		Visual scanning training with reading exercises. Non-Text: 300 Arabic digit-words, that were variable in length and comprise a beginning and end (length similar to 3–13 letters words, 10 units of 30 words, 45 min total), were read during one session. Participants were instructed to perceive each word as a whole before reading it aloud by shifting their gaze from the centre to the blind hemispace. An average of 10 sessions within 2 weeks.
[18]	HH = 9	78	60 (14)	2	3	RT = 1, RB = 1, LT = 1, LB = 1	8–30 Days^c^	Search for all targets defined by a specified feature (e.g., colour/form/conjunction) among distractors presented on a screen	Search duration was used as a performance measure in the correlation analysis.	
	UV = 9	nm	53 (14)							
[23]	HH = 4	75	54 (18)	0	4	0	11–22 Months^c^	Searching the letter A amongst 64 other letters of the alphabet presented on a screen		
	UV = 7	43	59 (3)							
[24]	HH = 4	75	54 (18)	0	4	0	11–22 Months^c^	Searching the letter A amongst 64 other letters of the alphabet presented on a screen		
	UV = 9	44	59 (3)							
[25]	HH = 10	80	50 (14)	4	6	0	6.4 months	Visual search number test. Search and identify the numbers 1 to 15 in ascending order		Audio–visual stimulation training. Participants were presented with three different types of sensory stimulations. 1. unisensory visual, 2. unisensory auditory, and 3. multisensory audio-visual. They had to perform visual explorations. When any visual stimulus was observed, they were asked to respond (button press). The training lasted 10 days (4h per day), and consisted of 30 blocks per day of 48 trials each.
[27]	HH = 6	50	65 (50–83)^b^	5	1	0	53 months (7–230)^b^	Detecting peripherally presented moving basketballs in a naturalistic virtual environment while sitting on a chair		
	UV = 6	66	63 (50–73)^b^							
[28]	HH = 8	63	62 (44–80)^b^	3	4	LB = 1	4–9wks^c^	Searching for differences between two almost identical black and white pictures presented next to each other horizontally on a screen		
	UV = 8	nm	25–71							
	HH = 5	40	61	2	2	LB = 1	5–9 Weeks^c^	Searching for differences between two almost identical black and white pictures presented next to each other horizontally on a screen		Visual search training. Computer-assisted training (45 min/day, 5 days per week, 4 weeks) consisting of visual search and basal cognitive functions.
[29]	HH = 14	64	57 (11)	8	6	0	7–180 Months^c^	The pop-out and serial exploration tasks were performed. In both exploration tasks, 63 images with 14, 24, or 48 balloons were presented. In the pop-out exploration task, participants had to find a balloon with a string amongst balloons without strings. In the serial exploration task, participants had to find the only balloon without a string among balloons with strings. In 1 out of 20 trials, the target was absent.		Visual scanning training. Adaptation training. The participants executed an anti-saccade in the direction opposite to the target in the visible hemispace. Upon completion of the anti-saccade, a feedback target was presented 10% deeper into the blind hemispace than the anti-saccade.
										Visual scanning training. Delayed shift training: The participants executed an anti-saccade in the direction opposite to the target in the visible hemispace. 800 ms after completion of the anti-saccade, a feedback target was presented 10% deeper into the blind hemispace than the anti-saccade.
										Visual scanning training. No shift training: The participants executed an anti-saccade in the direction opposite to the target in the visible hemispace. Upon completion of the anti-saccade, a feedback target was presented at the mirror position of the first presented target
[31]	HH = 12	67	22–6^c^	6	6	0	nm	Identify the numbers 1–15 that are positioned randomly in ascending order and are presented on a screen		
	UV = 12	42	40^e^							
[32]	HH = 23	65	50 (17)	13	10	0	nm	Searching for a small grey star within an everyday scene (e.g., landscape, building, edibles) with a small grey triangle as distractor		
	UV = 100	40	35 (11)							
			(44–72)^b^							
[34]	HH = 31	47	47 (24–75)^b^	13	18	0	≥3 Months	Search for a single randomly positioned target line, oriented either at 15ᴼ, 30ᴼ, or 45ᴼ amongst 40 vertical distractors.		Visual search training. Search for a target that differs from 40 homogenous distractors by a single feature (size or orientation). Training consist of 600 trials per 40 min session with 5 daily sessions per week during a month.

nm: not mentioned; HPH: high-performing people with homonymous hemianopia; LPH: low-performing people with homonymous hemianopia; HH: homonymous hemianopia; UV: people with unimpaired vision.

^a^RT: right top; RB: right bottom; LT: left top; LB: left bottom; ^b^mean (range); ^c^range.

**Table 3.** Performance-related scanning behaviour in search tasks.

	Reference	[8]	[18]^b^
	Reference system	GiW	EiH
	Head fixed	Yes	Yes
Number of events	Number of fixations	***HPH < LPH	*r* = 0.8***^c^
Repetitions	Percentage of item refixations	***HPH < LPH	–
	Percentage of item refixations in hemispace blind	**HPH < LPHR^a^	–
	Percentage repetition of scanpath	***HPH < LPH	–
Duration of events	Fixation duration	ns	–
Length of scans	Scanpath length	**HPH < LPH	–
	Mean saccadic amplitude directed towards hemispace blind	ns	–

**p < 0.01; ***p < 0.001; ns: not significant; – no results reported.

HPH: high-performing people with homonymous hemianopia; LPH: low-performing people with homonymous hemianopia; EiH: eye-in-head; GiW: gaze-in-wild.

^a^Low-performing people with right hemianopia; ^b^correlation analysis; ^c^indicating that a higher number of fixations relates to a higher performance.

**Table 4.** Spontaneous adaptations in scanning behaviour in search tasks.

	Reference	[2]	[18]	[23]	[27]	[28]	[31]	[32]
	Reference system	GiW	EiH	EiH	EiH	EiH	GiW	GiW
	Head restrained	Yes	Yes	Yes	No	Yes	Yes	Yes
Number of events	Number of fixations total	*HH > UV	–	*HH < UV	–	ns	*HH > UV	ns
	Number of saccades total	–	***HH > UV	–	–	–	–	–
	Fixations on environ-mental objects^a^	–	–	–	ns	–	–	–
Repetitions	Percentage of total item refixation	–	***HH > UV	–	–	–	*HH > UV	–
Duration of events	Fixation duration total	ns	ns	ns	*HH < UV	ns	ns	ns
	Saccadic duration	–	–	–	–	–	*HH > UV	–
Length of scans	Scan path length	*HH > UV	–	–	–	**HH < UV	*HH > UV	–
	Mean saccadic amplitude	n.s.	*HH < UV	–	–	*HH < UV	*HH < UV	**HH < UV
Area distribution of scanning	Horizontal area’s distribution of fixations	–	–	*HH > UV^b^	–	–	–	–
	Horizontal area’s distribution of fixation duration	–	–	*HH > UV^b^	–	–	–	–
	Percentage of fixation asymmetry between blind and visible hemispace	–	–	–	*HH > UV	–	–	–
	Horizontal area’s distribution of saccadic direction	–	–	ns	–	–	–	–
	Vertical area’s fixation distribution^c^	–	–	ns	ns	–	–	n.s.
	Vertical area’s fixation duration distribution^c^	–	–	ns	–	–	–	–
Scanning span	Mean fixation depth in hemispace blind	–	–	–	**HH > UV	–	–	–
Dispersion of scanning	Horizontal SD of fixations	–	–	–	*HH < UV	–	–	–
	Horizontal SD of head movements	–	–	–	ns	–	–	–
Scanning pattern	Scanning pattern	–	ns	–	–	–	–	–

*p < 0.05; **p < 0.01; ***p < 0.001; ns: not significant; – : no results reported; EiH: eye-in-head; GiW: gaze-in-world; HH: homonymous hemianopia; UV: people with unimpaired vision.

^a^Fixations on Sawhorse, ground, wall, lamppost, and others (murals, columns, stairs, windows, doors, etc.); ^b^difference in distribution in the far blind area of the 4 area’s (e.g., far-blind, near-blind, near-visible, far-visible hemispace); ^c^vertical distribution of fixations by dividing the environment in 2–4 upper and lower quadrants/quartiles.

**Table 5.** Training-induced alteration in scanning behaviour in search.

	Reference	[8]	[13] Text	[13] Non-text	[25]	[28]	[29] Adaptation training	[29] Delayed-shift training	[29] No-shift training	[34]
	Reference system	GiW	GiW	GiW	GiW	EiH	GiW	GiW	GiW	GiW
	Head fixed	Yes	Yes	Yes	Yes	Yes	Yes	Yes	Yes	Yes
Number of events	Number of fixations	***U > T^b^	ns	**U > T	*U > T	–	–	–	–	***U > T
	Number of initial fixations in incorrect hemispace before switching to other hemispace	–	–	–	–	–	–	–	–	*U > T
	Number of gaze shifts	^–^	–	–	–	–	–	–	–	***U > T
Repetitions	Percentage of item refixations	***U > T^a^	ns	ns	–	–	–	–	–	–
	Percentage repetition of scan path	***U > T^b^	–	–	–	–	–	–	–	–
Duration of events	Fixation duration	ns	ns	ns	–	–	ns	ns	ns	ns^c^
	latency of initial saccade	–	–	–	–	–	–	–	–	ns
	Angular velocity	–	–	–	–	–	–	–	–	***U > T
Length of scans	Scan path Length	***U > T^a^	–	–	–	ns	^–^	^–^	^–^	–
	saccade amplitude	**U < T	ns	ns	–	–	ns^d^	*U > T^e^	ns	ns
	Initial saccade amplitude	–	–	–	–	–	–	–	–	*U > T
Area distribution of scanning	Proportion of fixations in blind hemispace^f^	***U > T	–	–	–		–	–	–	ns
	Proportion of (initial) saccades directed towards blind hemispace^f^	–	–	–	–	–	–	–	–	ns
	Proportion fixations in upper compared to lower half of stimuli	–	–	–	–	ns	–	–	–	–

*p < 0.05; **p < 0.01; ***p < 0.001; ns: not significant; – : no results reported; EiH: eye-in-head; GiW: gaze-in-world; U: untrained; T: trained.

^a^Only for percentage refixations in blind hemispace; ^b^difference in both blind and visible hemispace; ^c^no significant difference for both blind and visible hemispace; ^d^only an increase in saccadic amplitude in the serial search task for people who adapted the length of saccades during training, but not for the whole training group; ^e^only in the pop-out search task in the blind hemispace; ^f^in percentage compared to visible hemispace.

#### Performance-enhancing scanning behaviour

Whether scanning behaviour affects performance can be determined by exploring the relationship between visual scanning behaviour and search performance. We found two studies that took this approach ([8,18]; Tables 2 and 3). One study compared high and low-performing people with HH in search tasks, based on search time [8]. The second study inferred performance-enhancing scanning behaviour from correlations between scanning behaviour and search time [18]. From these two studies we can deduce the following.

*Number of events.* In both studies, a lower number of fixations related to higher performance ([8,18]; Table 3). *Repetitions.* In one study, fewer repetitions of fixations or scanpaths related to higher performance [8]. *Duration of events.* In one study, fixation duration did not relate to performance [8]. *Length of scans.* In one study, a shorter scanpath related to higher performance, but the saccadic amplitude directed towards the blind hemispace did not relate to performance [8].

#### Spontaneous adaptation in scanning behaviour

To determine the presence of spontaneous adaptations to scanning behaviour in search tasks, nine articles ([2,8,18,23,24,27,28,30–32], see Tables 2 and 4) compared behaviour in people with HH to that in people with unimpaired vision. Since the same participant groups and results were reported in references [23] and [24], and in references [18] and [30], only the first article of each of these two authors is included.

*Number of events.* Three out [2,18,31] of six studies showed that people with HH make more fixations or saccades than people with unimpaired vision (Table 4). One of six studies showed the opposite results [23], while two studies did not show any difference in fixations between groups [28,32]. One study showed that people with HH make similar amount of fixations on environmental objects as people with unimpaired vision [27]. *Repetitions.* Two studies showed that people with HH more often visit the same fixation locations than people with unimpaired vision [18,31]. *Duration of events.* In six out of seven studies, people with HH show similar fixation durations as people with unimpaired vision [2,18,23,28,31,32]. In the other study, people with HH show shorter fixation durations than people with unimpaired vision [27]. One study showed that people with HH have longer saccadic durations [31]. *Length of scans.* In three articles, people with HH show a longer scanpath length than people with unimpaired vision [2,28,31]. In addition, in four out of five articles, people with HH show smaller saccades than people with unimpaired vision [18,28,31,32]. In the other study, people with HH showed similar saccadic amplitudes as people with unimpaired vision [2]. *Area distribution of scanning.* In two studies, people with HH fixate more frequently on their blind hemispace than people with unimpaired vision [23,27], specifically the far blind hemispace. Yet, in one study, no difference was found in saccadic direction [23]. Three studies did not find differences in vertical area distribution of fixations [23,27,32]. *Scanning span.* In one study, people with HH showed a higher mean scanning span into the blind hemispace (i.e., distance between the centre and the mean fixation position) than the mean scanning span into the hemifield with most fixations in people with unimpaired vision [27]. *Dispersion of scanning.* One study found that people with HH showed a smaller horizontal dispersion (i.e., horizontal SD) of fixations compared to people with unimpaired vision, but no difference in the horizontal dispersion of head movements [27]. *The pattern of scanning.* The single study that evaluated scanning patterns did not find any difference between people with HH and unimpaired vision [18].

#### Training-induced adaptations in scanning behaviour

Six studies have reported on the training-induced adaptations in scanning behaviour in search tasks ([8,13,25,28,29,34]; see Tables 2 and 5). Training-induced adaptations are changes in scanning behaviour due to training that can both occur consciously and unconsciously (for definitions see Supplementary Material Appendix D). An example of such a training-induced adaptation in scanning behaviour is making longer saccades after training compared to before training. These six studies report on a total of nine different training methods, consisting of one visual scanning training with search tasks, two visual scanning training with reading tasks, one audio–visual stimulation training, three visual scanning training, and two studies used visual search training (for definitions see Supplementary Material Appendix D). In most studies, search performance was defined in terms of search time [8,13,25,29,34]. One study did not report any performance measure before and after training [28], hence it is unclear whether the training improved performance. Performance was improved by four training methods [8,25,29,34], but the two training methods used in one study did not show any improvements [13]. In addition, two training methods (i.e., adaptation training and no-shift training) increased search performance in only one out of the two search tasks [29].

*Number of events.* Four out of five training methods reduced the number of fixations in search tasks [8,13,25,34], which also includes one training method that did not improve search performance ([13]; Table 5). The other training method did not alter the number of fixations [13]. In addition, one training method decreased the number of gaze shifts and a number of initial fixations in an incorrect hemispace before switching to another hemispace [34]. *Repetitions.* One out of three training methods, which increased search performance, reduced repetitions [8]. Two out of three training methods, which did not improve search performance, did not show a change in repetitions after training [13]. *Duration of events.* Seven training methods did not change the duration of fixations [13,29,34]. One training method did reduce the peak angular velocity of eye movements, but it did not affect fixation durations [34]. *Length of scans.* One out of two training methods reduced the scanpath length [8]. The other training method did not alter the scanpath length [28]. Only one out of seven training methods increased saccadic length [8]. Five out of seven training methods did not change the saccadic length [13,29,34], and one reduced the initial saccadic amplitude [34]. One training method reduced the saccadic length [29]. *Area distribution of scanning.* One out of two training methods increased the proportion of fixations in the blind hemispace [8]. The other training method did not change the proportion of fixations in the blind hemispace [34]. One training method, did not alter the proportion of fixations in the upper hemispace [28].

#### Summary of scanning behaviour in search tasks

The articles on performance-enhancing scanning behaviour in search tasks suggest that fewer fixations, fewer repetitions, and a shorter scanpath are related to better search performance. Spontaneous adaptations in scanning behaviour seem to result in more repetitions of scanning, shorter saccades, more frequent scanning of the blind hemispace, and scans further into the blind hemispace. Most training programs result in a reduction in the number of fixations. Training methods also seem to decrease repetitions, decrease the scanpath length, and increase scanning of the blind hemispace. One training method could also increase saccadic amplitude. Note that these alterations in scanning behaviour were not found for all training methods. Training that did not improve search performance did not alter the repetitions of scans, but seemed to reduce the number of fixations.

### Reading tasks

#### Performance-enhancing scanning behaviour

Only one article reported on performance-enhancing scanning behaviour during reading tasks ([40]; see Tables 6 and 7). Performance-enhancing scanning behaviour was determined by correlating various scanning parameters and reading time [40]. If available, the results are described separately for people with left-sided and right-sided HH, since they may experience different types of reading problems [41].

**Table 6.** Descriptives of article reporting scanning behaviour during reading.

Article	*N*	Gender (%M)	Age (M (SD))	Right-sided HH	Left-sided HH	Quadrant-anopia^a^	Time since onset (M (SD))	Task	Group division based on performance or used performance measure for correlation/regression analysis	Training
[13]	HH = 20	85	59 (12)	6	6	RT = 3, RB = 1	28.9 (28.4)	Participants silently read one out of three parallel versions of standardized reading text (61 words, 9 lines) at each time point (before training, after training and follow-up). Texts were sensitive to changes in oculomotor reading measures during treatment.		Visual scanning training with reading exercises. Text training: 300 single words (3–13 letters, 10 units of 30 words, 45 min total) were read during one session. Participants were instructed to perceive each word as a whole before reading it aloud by shifting their gaze from the centre to the blind hemispace. An average of 11 sessions within 2 weeks.
										Visual scanning training with reading exercises. Non-text training: 300 Arabic digit-words, that were variable in length and comprise a beginning and end (length similar to 3 to 13 letters words, 10 units of 30 words, 45 min total), were read during one session. Participants were instructed to perceive each word as whole before reading it aloud by shifting their gaze from the centre to the blind hemispace. An average of 10 sessions within 2 weeks.
[24]	HHL = 4	75	54 (18)	0	4	0	11–22 Months^c^	Subjects had to read two paragraphs that are well suited for adult readers. Only the first six lines (paragraph 1) and eight lines (paragraph 2) were read by PA-group and used for analysis.		
	UV = 7	43	59 (3)							
[29]	HH = 14	64	57 (11)	8	6	0	7–180 Months	Participants had to silently read two texts (85–90 words) extracted from French newspapers. A new text was presented at each session and visit.		Visual scanning training. Adaptation training: The participants executed an anti-saccade in the direction opposite to the target in the visible hemispace. Upon completion of the anti-saccade, a feedback target was presented 10% deeper into the blind hemispace than the anti-saccade.
										Visual scanning training. Delayed shift training: The participants executed an anti-saccade in the direction opposite to the target in the visible hemispace. 800 ms after completion of the anti-saccade, a feedback target was presented 10% deeper into the blind hemispace than the anti-saccade.
										Visual scanning training. No-shift training: The participants executed an anti-saccade in the direction opposite to the target in the visible hemispace. Upon completion of the anti-saccade, a feedback target was presented at the mirror position of the first presented target
[31]	HHL = 6	67	22–65^c^	0	6		5 Months − 30 years^c^	Participants read aloud 4 short stories (330 syllables)		Audio visual stimulation training. Audio-visual training. Participants were asked to detect the presence of visual targets, which were either presented alone or together with an acoustic stimulus
	HHR = 6			6	0					
	UV = 12	42	40^d^							
[36]	HHL = 4	75	54 (18)	0	4	0	11–22 Months^c^	Participants read two sets of 15 words consisting of an even amount of 4-letter, 5-letter, and 6-letter words. In all words the omission of the left letter would give rise to another English word.		
	UV = 9	44	59 (3)							
[37]	HHR = 18	67	24–73^c^	18	0		6 Months −14 years^c^	10 Text passages (∼50 words) from newspaper journalism were used. People with HHR read silently 3 of the 10 passages. People with UV read all 10 passages.		
	UV = 10	60	49–75^c^							
[38]	HHR = 6	67	54 (29)	4	0	RT = 1	14 (32) Weeks^e^	Four articles (word range 43–52, 7 lines, total of 194 words) from a local German newspaper were read silently.		
	UV = 6	67	56^d^							
[39]	HH = 11	55	nm	9	0	0	1 − 37 Years	Participants read 10 short text passages (approx. 50 words each) extracted from newspaper journalism at each of the time points.		Text reading training. Two blocks of moving text training. Participants read text from Sherlock Holmes stories, that horizontally moved from blind to seeing hemispace.
[40]	HHL = 25	56%	43 (21–64)^b^	0	25	0	5.8 Weeks (3–12)^b^	Participants had to silently read a text (61 words, 9 lines). Highly unfamiliar of foreign words were avoided.	Reading time was used as a performance parameter to correlate with visual scanning parameters.	Visual scanning training with reading exercises. Participants with left-sided HH had to read a text that slowly moved from the right to the left, so that they had to shift their gaze to the beginning of every word in a line of text (8–16 sessions). Participants with right-sided HH were instructed to not read a word before shifting their gaze to the end of the word (9-29 sessions).
	HHR = 25		38 (18–56)^b^	25	0	0				
	UV = 25	62.5%	38 (19–57)^b^				5.9 Weeks (4–9)^b^			

nm: not mentioned; HPH: high-performing people with homonymous hemianopia; LPH: low-performing people with homonymous hemianopia; HH: homonymous hemianopia; UV: people with unimpaired vision.

^a^RT: right top; RB: right bottom; LT: left top; LB: left bottom; ^b^mean (range); ^c^range; ^d^mean.

**Table 7.** Performance-related scanning behaviour in reading tasks.

	Reference	[40]	
	Reference system	GiW	
	Head fixed	Yes	
	Groups	HHL	HHR
Number of events	Number of fixations	*r* = 0.67*	*r* = 0.82*
	Number of saccades towards directed blind hemispace	ns	ns
	Number of saccades towards directed the visible hemispace	*r* = 0.64*	ns
Repetitions	Repetition of fixations	*r* = 0.71*	*r* = 0.67*
	Repetition of saccades directed towards the blind hemispace	*r* = 0.60*	ns
	Repetition of saccades directed towards the visible hemispace	ns	ns
Duration of events	Fixations duration	*r* = 0.66*	*r* = 0.79*
Length of scans	Amplitude of saccades directed towards the blind hemispace	*r*= −0.52*	*r*= −0.64*
	Amplitude of saccades directed towards the visible hemispace	ns	n.s.

*p < 0.05; ns: not significant; – : no results reported; EiH: eye-in-head; GiW: gaze-in-world; HHR: homonymous hemianopia right; HHL: homonymous hemianopia left.

*Number of events.* In people with HH, a lower number of fixations related to higher performance ([40]; Table 7). In people with left-sided HH, a lower number of saccades towards the visible hemispace is related to higher performance. *Repetitions.* In people with HH, less repetitions related to a higher performance. In people with left-sided HH, less repetitions of saccades to the blind hemispace related to higher performance. *Duration of events.* In people with HH, shorter fixation durations are related to higher performance. *Length of scans.* In people with HH, longer saccades towards the blind hemispace relates to higher performance.

#### Spontaneous adaptation in scanning behaviour

Six studies focussed on spontaneous adaptations in scanning behaviour during reading tasks ([24,31,36–38,40]; see Tables 6 and 8). In addition, people with left-sided and right-sided HH were compared with each other.

**Table 8.** Spontaneous adaptations in scanning behaviour in reading tasks.

	Reference	[24]	[31]	[36]	[37]	[38]	[40]
	Reference system	EiH	GiW	EiH	EiH	GiW	GiW
	Head fixed	Yes	Yes	Yes	No	Yes	Yes
	HH groups	HHL	HHL & HHR	HHL	HHR	HHR	HHL & HHR
Number of events	Number of fixations	ns^a^	^–^	ns	***HHR > UV	**HHR > UV^a^	*HH > UV
	Number of saccades directed towards blind hemispace	ns	***HHR > UV HHL: ns	–	–	–	*HH > UV
	Number of saccades directed towards visible hemispace	–	***HHR > UV HHL: ns	–	–	ns	ns
	Number of return to sweep saccades	–	HHR: ns ***HHL > UV	–	–	–	–
Repetitions	Percentage of re-fixations	–	–	–	***HHR > UV	–	*HH > UV^b^
	Percentage of saccadic repetitions towards blind hemispace						*HHR > UV
Duration of events	Fixation duration	ns	**HHR > UV	–	***HHR > UV	**HHR > UV	*HHR > UV HHL: ns
Length of scans	Saccadic amplitude	–	***HHR < UV	–	–	–	–
	Saccadic amplitude directed towards blind hemispace	–	–	–	***HHR < UV	*HHR < UV	*HH < UV
	Amplitude return to sweep saccades	–	–	–	–	ns	–
	Saccadic amplitude directed towards the visible hemispace	–	–	–	*HHR < UV	ns	HHR: ns *HHL < UV
Area distribution of scanning	Proportion of fixation duration in the blind hemispace	–	–	ns	–	–	–
Scanning span	Initial landing position to the left	–	–	–	HHR > UV	–	–

*p < 0.05; **p < 0.01; ***p < 0.001; ns: not significant; – : no results reported; EiH: eye-in-head; GiW: gaze-in-world; HH: homonymous hemianopia; HHL: homonymous hemianopia left; HHR: homonymous hemianopia right; UV: people with unimpaired vision.

^a^Fixations per word or character; ^b^difference only for the blind hemispace.

*Number of events*. In three studies, people with right-sided HH showed more fixations than people with unimpaired vision ([37,38,40]; Table 8). In one out of three studies, people with left-sided HH showed more fixations compared to people with unimpaired vision [40]. In the other two study, people with left-sided HH showed a similar number of fixations as people with unimpaired vision [24,36]. In two articles, people with right-sided HH showed more saccades directed towards the blind hemispace compared to people with unimpaired vision [31,40]. In one out of three articles, people with left-sided HH showed more saccades directed towards the blind hemispace compared to people with unimpaired vision [40]. In the other two studies, people with left-sided HH showed a similar amount of saccades directed towards the blind hemispace as people with unimpaired vision [24,31]. In one out of three articles people with right-sided HH showed more saccades directed towards the visible hemispace compared to people with unimpaired vision [31]. The other two studies did not show a difference between people with right-sided HH and unimpaired vision [38,40]. Two studies also did not show this difference for people with left-sided HH compared to unimpaired vision [31,40]. In one article, people with left-sided HH show more return to sweep saccades compared to people with unimpaired vision, but people with right-sided HH showed a similar amount of return to sweep saccades as people with unimpaired vision [31]. *Repetitions.* In one study people with left-HH make more repetitions of fixations than people with unimpaired vision [40]. In two studies, people with right-sided HH make more repetitions of fixations than people with unimpaired vision [37,40]. In one study, people with right-sided HH showed more repetitions of saccades directed towards their blind hemispace compared to people with unimpaired vision [40]. *Duration of events.* In four studies, people with right-sided HH showed longer fixations durations than people with unimpaired vision [31,37,38,40]. In two studies, people with left-sided HH did not differ in fixation duration compared to people with unimpaired vision [24,40]. *Length of scans.* In one study, people with right-sided HH showed shorter saccadic amplitudes than people with unimpaired vision [31]. In three studies, people with right-sided HH show shorter saccades towards their blind hemispace compared to unimpaired vision [37,38,40]. In one study, people with left-sided HH also showed shorter saccades towards their blind hemispace compared to unimpaired vision [40]. One article showed no difference in the length of return to sweep saccades between people with right-sided HH and unimpaired vision [38]. In one out of three studies, people with right-sided HH show shorter saccades towards their visible hemispace compared to unimpaired vision [37]. The other two studies did not find a difference between people with right-sided HH and unimpaired vision in length of saccades towards the visible hemispace [38,40]. In one study, people with left-sided HH also show shorter saccades towards their visible hemispace compared to unimpaired vision [40]. *Area distribution of scanning.* In one article, people with HH did not differ from people with unimpaired vision [37]. *Scanning span.* In one study, people with right-sided HH showed a more leftward landing position in seven-letter words than people with unimpaired vision [37].

#### Training-induced adaptations in scanning behaviour

Studies have also addressed how training methods can adapt scanning behaviour, such as the number of saccades towards the blind visual field. We call this as training-induced adaptations in scanning behaviour. Five studies have reported on training-induced adaptations in scanning behaviour in reading tasks ([13,29,31,39,40]; Tables 6 and 9), containing eight training methods in total. These methods consisted of three visual scanning training with reading exercises, three visual scanning training, one audio–visual stimulation training, and one text reading training (for definitions see Supplementary Material Appendix D). Reading performance was improved by all training methods [13,29,31,39,40]. However, the delayed-shift and no-shift training method only improved reading performance in people with right-sided HH, but not left-sided HH [29].

**Table 9.** Training-induced alteration in scanning behaviour during reading.

	Reference	[13] Text	[13] Non-text	[29] Adaptation training	[29] Delayed-shift training	[29] No-shift training	[31]	[39]	[40]
	Reference system	GiW	GiW	GiW	GiW	GiW	GiW	EiH	GiW
	Head fixed	Yes	Yes	Yes	Yes	Yes	Yes	–	Yes
	HH groups	HHR & HHL	HHR & HHL	HHR & HHL	HHR & HHL	HHR & HHL	HHR & HHL	HHR	HHR & HHL
Number of events	Number of fixations	HH: *U > T	HH: *U > T	–	–	–	–	HHR: ***U > T	HH: **U > T
	Number of forward / progressive saccades	HH: *U > T	HH: *U > T	HH: ns	HH: ns	HH: ns	–	–	HH: **U > T
	Number of backward / regressive saccades	–	–	–	–	–	HHR: *U > T HHL: ns	HHR: ns	HH: **U > T
	Number of return to sweep saccades	–	–	HH: ns^a^	HHR: *U > T HHL: ns	HHR: *U > T HHL: ns	HHR: ns HHL: **U > T	–	–
Repetitions	Percentage of refixations	HH: *U > T	HH: *U > T	–	–	–	–	HHR: ***U > T	HH: **U > T
	Repetitions of saccades								HHR: *U > T^b^ HHL: ns
Duration of events	Fixation duration	HH: *U > T	HH: *U > T	HH: ns	HH: ns	HH: ns	HHR: *U > T HHL: ns	HHR: ns	HHR: **U > T HHL: ns
Length of scans	Scanpath length	HH: *U > T	HH: *U > T	–	–	–	–	–	–
	Saccadic amplitude	HH: *U < T	HH: *U < T	–	–	–	HHR: *U < T HHL: ns	–	–
	Amplitude forward / progressive saccades	–	–	HH: ns	HH: ns	HH: ns	–	HHR: ***U < T	HHR: **U < T HHL: ns
	Amplitude backward/regressive saccades	–	–	–	–	–	–	–	HH: **U > T
	Amplitude return to sweep saccades	–	–	HH: ns^a^	HH: ns	HH: ns^a^	–	–	–
	Amplitude saccade that produces the first fixation within a word (incoming saccadic amplitude)	–	–	–	–	–	–	HHR: **U < T	–
Scanning span	Initial landing position to left	–	–	–	–	–	–	HHR: ns	–

*p < 0.05; **p < 0.01; ***p < 0.001; ns: not significant; – : no results reported; EiH: eye-in-head; GiW: gaze-in-world; U: untrained; T: trained; HH: homonymous hemianopia; HHL: homonymous hemianopia left; HHR: homonymous hemianopia right.

^a^Only a significant increase in people with HHL who adapted their saccadic length during training, but not the whole training group; ^b^for people with HHR, reduction in repetitions of saccades to the left and right hemispace.

*Number of events.* Four training methods have been shown to reduce the number of fixations in people with right-sided HH ([13,39,40]; Table 9). Three training methods reduced the number of fixations in left-sided HH [13,40]. Three out of six training methods reduced the number of forward saccades in both people with right and left-sided HH [13,40]. The other three training methods did not alter the number of forward saccades [29]. Two out of three training methods decreased the number of regressive saccades in people with right-sided HH [31,40]. The other training methods did not change the number of regressive saccades [39]. One out of two training methods decreased the number of regressions in people with left-sided HH [40]. The other training method did not change the number of regressions [31]. Two out of four training methods reduced the number of returns to sweep saccades in people with right-sided HH [29]. The other two studies did not alter the number of returns to sweep saccades [29,31]. One out of four training methods reduced the number of returns to sweep saccades in left-sided HH [31]. The other three studies did not show this change [29]. *Repetitions.* Four training methods decreased the number of fixation repetitions in people with HH [13,39,40]. One training method decreased the repetitions of saccades towards the left and right hemispace in only people with right-sided HH [40]. *Duration of events.* Four out of eight training methods reduced the fixation duration in people with right-sided HH [13,31,40] and two out of seven reduced the fixation duration in people with left-sided HH [13]. The other training methods did not alter fixation durations [29,31,39,40]. *Length of scans.* Two training methods reduced scanpath length in people with HH [13]. Three training methods increased saccadic amplitude in people with right-sided HH [13,31], and two out of three increased saccadic amplitude in people with left-sided HH [13]. One training method did not alter saccadic amplitude in left-sided HH [31]. Two out of five training methods increased the length of forward saccades in people with right-sided HH [39,40]. The other three training methods did not alter the length of forward saccades [29] and none of the four training methods alter the length of forward saccades in people with left-sided HH [29,40]. One training method increased the length of regressive saccades in people with HH [40]. Three training methods did not alter the length of return to sweep saccades in people with HH [29]. One training method induced a longer first saccade within a word in people with right-sided HH [39]. *Scanning span.* The only study that reported on the scanning span did not show any change after training [39]

#### Summary of scanning behaviour in reading tasks

In reading tasks, high performance seems to be related to a relatively low number of fixations, few repetitions, short fixation durations, and long saccades towards the blind hemispace. People with left-sided HH may also benefit from making less saccades towards their visible hemispace to increase reading performance. In contrast, spontaneous adaptations in scanning behaviour for both people with left- and right-sided HH appear to be characterised by relatively many repetitions, and short saccades directed towards the blind hemispace. People with right-sided HH specifically may also spontaneously show relatively many fixations, many progressive saccades, long fixations durations, and a landing position of fixations distributed further into the visible hemispace, compared to people with unimpaired vision. People with left-sided HH tend to spontaneously show more return to sweep saccades. Training was reported to reduce the number of fixations, decrease the number of forward saccades, reduce fixation repetitions, reduce saccadic repetitions, increase the length of saccades, and decrease the duration of fixations. Note that these alterations in scanning behaviour were not found for all training methods.

### Mobility tasks

#### Performance-enhancing scanning behaviour

Six studies reported performance-enhancing scanning behaviour in mobility tasks ([48,49,53–56]; see also Tables 10 and 11). Five of these six studies involved in driving [49,53,54,56,57], and one involved walking [55]. We will accentuate whether the outcomes reported involve driving or walking studies because the performed scanning behaviour may differ between these tasks. All studies, excluding one [49], examined the difference between high and low-performing people with HH. Group division was based on various performance parameters (see also Table 10). Two studies based their group division on passing or failing the driving test when driving on-road [54,56]. The other studies based the group division on, respectively, the number of undetected hazards, average time per correctly collected item, and number of collisions [53,55,57]. A regression analysis was performed by two studies [49,57] with performance defined by (early) detection of hazards.

**Table 10.** Descriptives of articles reporting scanning behaviour during mobility.

Article	*N*	Gender (%)	Age (M (SD))	Right-sided HH	Left-sided HH	Quadrant- anopia^a^	Time since onset (M ± SD)	Task	Group division based on performance or used performance measure for correlation/regression analysis
[27]	HH = 6	50	65 (50–83)^b^	5	1	0	53 months (7–230)^b^	Detecting peripherally presented moving basketballs in a naturalistic virtual environment while walking	
	UV = 6	66	63 (50–73)^b^						
[48]	HH = 16	81%	54 (39,61)^d^	5	11	RT or LT = 1	3.5 (2, 9.5)^e^	Driving on a virtual road. Participants had to stop for pedestrians who approach intersections to cross the street.	
	UV = 16	63%	50 (39,66)^d^						
[49]	HH = 17	88	56 ± 20	8	9	0	4.7 years (0.8 − 13)^b^	Driving on a virtual road. Motorcycles appear at the intersections. Participants needed to press a horn when they detected a motorcycle. Participants drove on the left lane	Performance measure is early detection, i.e., the detection of the motorcycle before driver reached the intersection
[52]	HH = 13	76.9%	LH = 54 (9); RH = 46 (15)	6	7	0	1.7–6.1 years^c^	Driving on road in a simulator in an urban environment with multiple intersections. Pedestrians need to be detected. Participants drove on the left lane.	
	UV = 12	75%	51 (13)						
[53]	HPH = 5	nm	nm	nm	nm	nm	nm	Crossing a virtual intersection while driving without causing a collision with vehicles approaching from the left and right side at two density levels, namely 50% and 75%.	Hemianopia participants were divided into the HPH and LPH group based on the number of collision by means of the median split method
	LPH = 9	nm	nm	nm	nm	nm	nm		
[54]	HPH = 4	75	56 (11)	1	1	RT = 1, LT = 1	4 (4) Years	Driving on road (37.5 km) in a simulator with 9 hazardous situations which needed to be prevented. A certified driving instructor rated each hemianopia participant on driving fitness. Participants drove on the left lane	HPH passed, LPH failed the driving fitness test.
	LPH = 4	25	48 (11)	1	2	LT = 1	10 (4) Years		
[55]	HPH = 7	nm	53 (13)	3	4	0	≥6 Months	Looking for and collecting 20 different products marked with an orange sign on shelves in a supermarket by walking through the corridor only once.	People with hemianopia included in the LPH group if their average time per correctly collected item was above the mean plus 3 times the SD of the unimpaired vision group. All other hemianopia subjects were included in the HPN group.
	LPH = 3	nm		1	2	0	≥6 Months		
[56]	HPH = 6	nm	53 (13)	4	2	0	≥6 Months	Driving test on-road (20 km) with a driving instruction, who examined driving performance. Participants drove on the left lane	LPH group were people with hemianopia that failed on fit to drive. People with hemianopia that passed to fit to drive were included in the HPH group
	LPH = 4	nm		0	4	0	≥6 Months		
[57]	HPH = 6	33	56 (17)	3	3		228 (112) Days	Driving on road in a simulator. Eight potentially hazardous objects need to be detected and collisions need to be avoided.	People with hemianopia in the HPH group missed <2 hazards and those in the LPH missed >2 hazards
	LPH = 8	63	58 (13)	4	4		630 (769) Days		

nm: not mentioned; HPH: high-performing people with homonymous hemianopia; LPH: low-performing people with homonymous hemianopia; HH: homonymous hemianopia; UV: people with unimpaired vision.

^a^RT: right top; RB: right bottom; LT: left top; LB: left bottom; ^b^mean (range); ^c^range; ^d^mean (interquartile range).

**Table 11.** Performance-related scanning behaviour in mobility tasks.

	Head fixed	[49]	[53]	[54]	[55]	[56]	[57]
	Reference system	EiH	GiW	EiH	EiH	EiH	EiH
	Head fixed	No	No	No	No	No	No
Number of events	Number of fixations	–	ns	–	–	–	–
	Number of saccades	–	–	–	–	–	ns
	Number of head movements	–	–	*HPH > LPH	–	–	ns
	Number of shifts between hemispaces	–	***HPH > LPH	–	–	–	–
	Number of fixation on task-relevant objects	–	***HPH > LPH	–	–	–	–
Duration of events	Fixation duration	–	ns	–	–	–	ns
	Saccadic duration	–	–	–	–	–	ns
	Peak velocity saccades	–	–	–	–	–	*HPH > LPH
	Duration of head movements	–	–	–	–	–	ns
	Peak velocity of head movements	–	ns	–	–	–	ns
Length of scans	Scan path length	–	***HPH > LPH	–	–	–	–
	Saccadic amplitude	***CI . 20–0.63^e^	–	*HPH > LPH	–	–	*HPH > LPH
	Head movements amplitude	***CI 11 – 0.61^e^	–	–	–	–	ns
Area distribution of scanning	Proportion of fixations or glances in hemispace blind	–	ns	–	+HPH > LPH^a^	ns	ns
	Proportion of glance duration in blind hemispace	–	–	–	ns^b^	ns	–
	Proportion of head movements directed towards blind hemispace	–	–	–	–	–	ns
	Proportion of central fixations or glances	–	*** HPH < LPH	–	–	ns	–
Scanning span	Glance depth from midline	–	***HPH > LPH^c^	–	–	–	–
Dispersion of scanning	Variance or SD of horizontal fixation or glance locations	–	–	–	ns	ns	** HPH > LPH^d^
	Variance of vertical fixation locations	–	–	–	–	–	ns

*p < 0.05; **p < 0.01; ***p < 0.001; + trend *p* < 0.1, ns: not significant; – : no results reported; EiH: eye-in-head; GiW: gaze-in-world; HPH: high-performing people with homonymous hemianopia; LPH: low-performing people with homonymous hemianopia.

^a^No significant difference when looking at the proportion of glances in hemispace beyond 60 or 30 degrees; ^b^only a significant difference in proportion of glances in hemispace beyond 60 degrees between HPH with left hemianopia and LPH; ^c^no significant difference for the blind hemispace; ^d^regression analysis also showed that the variance of fixations predicted mobility performance (**R^2^=0.41); ^e^determining prediction of performance by repeated train and test approach with Matthews correlation coefficient and the confidence interval (CI).

*Number of events.* Only studies that involved driving reported the number of events. In two studies, the number of fixations and saccades did not relate to performance ([53,57]; Table 11). In only one out of two studies, more head movements were related to higher performance [54]. The other study did not find such a relation [57]. One study found that many shifts between the blind and visible hemispaces and many fixations on task-relevant information relate to high performance [53]. *Duration of events.* Only studies that involved driving reported on the duration of events. In one study, saccadic duration, duration of head movements, and peak velocity of head movements did not relate to high performance [57]. Only the peak velocity of saccades was related to high performance, but shorter saccadic duration did not [57]. In addition, two studies did not find a relation between fixation duration and performance [53,57]. *Length of scans.* Only studies that involved driving reported on the length of scans. In one article, a larger scanpath length was related to higher performance [53]. In three studies, larger saccadic amplitudes were related to higher performance [49,54,57]. In one out of two studies, a larger length of head movements was related to higher performance [49]. The other study did not find such a relation [57]. *Area distribution of scanning.* Area distribution of scanning was reported in both the studies that involved driving and the study that involved walking. In three out of four studies, the proportion of scanning the blind hemifield was not related to high performance [53,56,57]. These were all studies that involved driving. In the study that involved walking, a trend was found indicating that a higher proportion of scanning the blind field was related to high performance, yet this was not significant [55]. In one out of two studies that involved driving, a lower distribution of scans in the central area was related to higher performance [53]. *Scanning span.* Only one study involving driving reported the scanning span. In one study, a larger scanning span into the visible hemispace was related to higher performance, because a larger absolute scan depth was related to high performance, but the scan depth into the blind hemispace did not show a relation to performance [53]. *Dispersion of scanning.* The dispersion of scanning was reported in both studies that involved driving and walking. In only one out of three studies, high horizontal dispersion of scans (e.g., variance, standard deviation) was related to high performance [57]. This was one of the two studies involving driving. In the other driving study and the walking study, no such relation was found [55,56]. In one study that involved driving, the vertical dispersion was not related to performance [57].

#### Spontaneous adaptation of scanning behaviour

Only three studies have focussed on spontaneous adaptation in scanning behaviour in people with HH in mobility tasks ([27,48,52]; Tables 10 and 12;). These mobility tasks were two studies that involved driving [48,52] and one study that involved walking [27].

**Table 12.** Spontaneous adaptations in scanning behaviour by number of events, duration of events, length of scans, and distribution of scanning during mobility tasks.

	Reference	[27]	[48]	[52]
	Reference system	EiH	GiW	–
	Head Fixed	No	No	No
Number of events	Fixations on environmental objects	ns^a^	ir.^b^	–
	Number of head movements	–	–	ns
	Percentage of not making head movements	–	–	ns
Duration of events	Fixation duration	* HH < UV	–	–
	Fixation duration on task-relevant objects	–	ir. ^c^	–
	Duration to the first fixation on task-relevant objects	–	ir.^d^	–
Length of scans	Amplitude of head movements	–	–	***HH < UV
	Amplitude of gaze scans directed towards visible hemispace		*HH > UV	
Area distribution of scanning	Percentage of head movements directed towards blind hemispace	–	–	**HH > UV
	Number of gaze scans directed towards visible hemispace		*HH < UV	
Scanning span	Mean fixation depth in hemispace blind	ns	–	–
Dispersion of scanning	Horizontal SD of fixations	ns	–	–
	Horizontal SD of head movements	ns	–	–

*p < 0.05; **p < 0.01; ***p < 0.001; ns: not significant; – : no results reported; ir.: inconclusive results; EiH: eye-in-head; GiW gaze-in-world; HH = homonymous hemianopia; UV = people with unimpaired vision.

^a^Fixations on sawhorse, ground, wall, lamppost, and others (murals, columns, stairs, windows, doors, etc.); ^b^in general, participants with UV and HH neither differ on fixation rates on pedestrians nor cars while driving in a simulator. There was one exception. Only when cars were approaching from one side, people with HH fixated less on cars than people with UV; ^c^the article reported different results. Fixation durations on cars approaching in the visible hemispace in people with HH were longer than people with UV when cars approach from both sides and the participants fixated on only one car. No difference was found in fixation duration when participants fixated on cars approaching from both directions, or when cars approached from one side only. In contrast, when fixating on pedestrians approaching in the visible hemispace, the fixation durations of HH were shorter than UV; ^d^only for first fixations on pedestrians approaching in the visible hemispace, but not on cars.

*Number of events.* Both studies that involved driving and walking reported the number of events. In one study that involved driving, people with HH did not differ in the number of head movements compared to unimpaired vision ([52]; Table 12). In one out of two articles, people with HH did not differ in fixations on environmental objects compared to unimpaired vision [27]. This study involved walking. In the study that involved driving, inconclusive results were reported [48]. *Duration of events.* Both studies that involved driving and walking reported the duration of events. In the study that involved walking, people with HH showed shorter fixation durations than people with unimpaired vision [27]. In one study that involved driving, inconclusive results were reported on the fixation duration and duration to first fixation on task-relevant objects [48]. *Length of scans.* Only studies that involved driving reported on the length of scans. In one article, people with HH show smaller head movements compared to people with unimpaired vision [52]. In one article, people with HH showed larger scans towards the visible hemispace compared to people with unimpaired vision [48]. *Are distribution of scanning.* Only studies that involved driving reported on the area distribution of scanning. In one article, people with HH showed a higher distribution of scans directed towards the blind hemispace compared to people with unimpaired vision [52]. In one article, people with HH made less scans towards the visible hemispace compared to people with unimpaired vision [48]. *Scanning span.* Only studies that involved walking reported on the scanning span. In this article, people with HH did not differ in scanning span compared to people with unimpaired vision [27]. *Dispersion of scanning*. Only the study that involved walking reported on the dispersion of scanning. In this article, people with HH and people with unimpaired vision did not differ on dispersion [27].

#### Training-related adaptations in scanning behaviour

No research has been found on training-induced adaptations in scanning behaviour in mobility tasks.

#### Summary of scanning behaviour in mobility tasks

Driving performance may be enhanced by relatively frequently shifting gaze between both hemispaces, focussing more on task-relevant information, scanning deeper into the visible hemispace, and having a longer length of eye scans. Walking performance might be enhanced by a higher distribution of scans in the blind hemispace and a larger dispersion of scanning. People with HH seem to spontaneously adopt one of these performance-enhancing scanning behaviour. They made larger scans into the visible hemispace during driving. The other spontaneous adaptations in scanning behaviour were not similar to these performance-enhancing scanning behaviour. They spontaneously showed short fixation durations in the walking task. In driving tasks, they showed smaller head movements, frequent scanning of the blind hemispace by head scans, and less frequent scanning of the visible hemispace. It should be mentioned that only three studies reported on the spontaneous adaptations in scanning behaviour of which one driving article showed several inconclusive results. Training-induced adaptations in scanning behaviour were not reported in any article.

## Discussion

In this systematic review, we evaluated which scanning behaviour enhances performance in different tasks in people with HH, and whether this scanning behaviour is similar to adaptations in scanning behaviour that occur spontaneously or can be induced by training. Overall, current evidence suggests that performance-enhancing scanning behaviour often differs between tasks, but some small similarities were found as well. For both search and reading tasks, higher performance was associated with people making fewer fixations and repetitions. In reading and mobility tasks, specifically driving, making longer saccades was beneficial for performance. In reading tasks, these relatively longer saccades have to be specifically directed towards the blind hemispace. In driving tasks, long scans should be directed to both hemispaces.

Another general conclusion that can be deduced is that, on average, people with HH do not spontaneously adopt task-specific performance-enhancing scanning behaviour, at least in reading and search tasks. Spontaneous adaptations often differ from or are even opposite to the behaviour that is associated with higher task performance. Training can, however, induce this performance-enhancing scanning behaviour. Therefore, compensatory scanning training aiming to improve reading and search performance may be beneficial for people with HH to reduce difficulties they experience in performing these tasks.

In the following section, we will elaborate on these conclusions. Current evidence shows that in search tasks, on the one hand, people with HH spontaneously make more repetitions and have longer scanpaths. Training, on the other hand, reduces the number of repetitions and scanpath length. Fewer repetitions and shorter scanpaths are associated with higher performance. Thus, this implies that spontaneous adaptations are generally ineffective, whereas adjustments to scanning behaviour acquired through training generally are effective. Comparable results are also found in reading tasks. Fewer repetitions and longer saccades towards the blind hemispace relate to higher performance, while people with HH spontaneously make more repetitions and saccades directed towards the blind hemispace are shorter. People with right-sided HH in particular spontaneously fixate longer and make many repetitions, while this behaviour is negatively associated with performance. Training could induce performance-enhancing scanning behaviour in terms of (i) a reduction in the number of fixations, (ii) a decrease in fixation duration, (iii) a reduction in the number of forward/regressive saccades, (iv) a reduction in fixation/saccadic repetitions, and an increase in the length of saccades. In mobility tasks, we found one similarity between scanning behaviour that improves performance and is adopted spontaneously. This scanning characteristic was longer scans into the visible hemispace and was found in studies involving driving. No other (dis)similarities could be found, due to the lack of reporting on identical scanning parameters between articles and the inconclusive results within articles. In addition, it is not possible to establish whether training-induced adaptations in mobility tasks are related to performance-enhancing scanning behaviour, since we did not find any articles reporting on these changes in scanning behaviour.

### Recommendations for future research into scanning behaviour

#### Knowledge gaps

The outcomes of this systematic review show a knowledge gap in the literature describing scanning behaviour in people with HH. Few articles reported on performance-enhancing scanning behaviour in search and reading tasks [8,18,40], only a few articles provided insights into spontaneous adaptation in scanning behaviour in mobility tasks [27,48,52], and no articles have reported on training-induced adaptations in scanning behaviour in mobility tasks. To accommodate future research trying to fill this knowledge gap, we will provide some suggestions for reporting the methods and results.

#### Reporting the task, task-performance parameters, and scanning behaviour parameters

Interpretation and comparison of results across different studies could be substantially improved by reducing variation in scanning behaviour parameters and methods for analysis. A previous study already proposed that a core set of clinically relevant scanning behaviour parameters should be defined and reported by researchers working on scanning behaviour in people with hemianopia [17]. To establish this core set of scanning behaviour parameters, we could follow the same study design as has been used to find a consensus on clinical relevant outcome parameters for visual functioning [77]. The usage of this core set should, however, not limit the development of new analyses for scanning behaviour. Lastly, the interpretation of outcomes could also be supported by a detailed method section on the participants, tasks, definition of gaze events, data analyses, and data quality (see [78] for some guidelines).

#### Reporting individual and subgroup data

Comparisons between groups may mask relevant outcomes on scanning behaviour in hemianopia, considering that the population of people who have acquired HH due to a brain injury is quite heterogeneous. People may have different comorbidities, such as a lower visual processing speed [79] or a reduced working memory due to their acquired brain injury [80]. These comorbidities could influence spontaneous adaptations of scanning behaviour. It is, for example, suggested that individuals with more physical and cognitive restrictions are less able to use compensatory strategies [81]. Comorbidities can also influence the effect of scanning behaviours that have been shown to improve performance. For example, people with a lower visual processing speed may need more time to obtain certain information from the environment. Even in the absence of HH, this could already lead to longer reaction times and fixations. A relation between time-dependent performance measures, such as search and reading time, and fixation durations may thus be modulated by visual processing speed. It is also suggested that a reduced working memory capacity could contribute to inadequate performance of people with HH when crossing streets while driving [82]. Even in the absence of HH, impaired working memory may result in repeated scanpaths or longer fixations on the same location in the environment to memorise the information necessary for collision avoidance and safe crossings. Due to different comorbidities, people might thus be less able to spontaneously adapt their scanning behaviour and may need to perform different scanning behaviour to improve task performance than the group average. More importantly, rehabilitation training based on these averages may thus not facilitate the highest outcomes in all individuals with HH or may even in some cases not facilitate any improvements at all.

Group averages may also hide the full spectrum of performance-enhancing scanning behaviour that could be used. For example, smaller saccades combined with larger head movements may be as effective as larger saccades with smaller head movements. If so, a group average would not reveal the actual relationship between performance and scanning behaviour. High-performing people with HH also vary considerably in scanning behaviour [26], suggesting that there might not be only one key set of performance-enhancing scanning behaviours. It is thus crucial to obtain more information on scanning behaviour in individuals, so we can create rehabilitation training tailored to the individual.

#### Reporting time-independent scanning parameters

Time-dependent parameters may not always provide an accurate description of scanning behaviour. For example, with an extended search time, more fixations can be made prior to task completion. Still, the number of fixations per minute may not differ from people with a shorter search time. Thus, describing relevant changes in the scanning behaviour of individuals requires reporting time-independent scanning parameters.

#### Reporting relations between scanning parameters

Finally, it should be considered that most outcomes of scanning parameters may depend on the outcomes of other scanning parameters. For example, many saccades in reading tasks could relate to shorter saccades because after a short saccade the next line or word might not fall into the visible hemispace. Therefore, a new saccadic jump must be performed. Another example is that high-performing people with HH may show few fixations in blind hemispace in search tasks because they make fewer repetitions of fixation. These relationships can be identified by correlating scanning parameters. For example, it has been found that the saccadic amplitude, peak velocity of saccades, and horizontal variance of eye movements relate to each other during driving on the road [57], implying that larger saccades might result in higher peak velocity and a larger dispersion of scanning.

### Training of scanning behaviour

Providing compensatory scanning training seems necessary for some people with HH to overcome problems with reading and search tasks. Our results suggest that at least some of the people with HH do not spontaneously apply scanning behaviour that is related to high performance. This could explain why people with HH report difficulties with reading and search tasks [3,4,6,8–10,40,42]. Our results also suggest that at least some of the compensatory scanning training programs induce scanning behaviour that improves performance. The promising results of the training methods could thus be caused by this performance-enhancing scanning behaviour [8,11–15]. Yet, there is still room for improvement. New task-specific instruction and feedback on scanning behaviour may be derived by filling the knowledge gaps. This review specifically aimed to address whether current training methods can induce scanning behaviour that improves performance, but it did not include training effects. The latest review on training effects of compensatory scanning training included four articles that compared a training group to a control group of which only one article reported changes in scanning behaviour ([16,39]). Randomized controlled trials could, thus, benefit from reporting changes in scanning behaviour because altered scanning behaviour can improve task performance and contribute to training effects.

Even though much research still needs to be performed, the current findings do provide some preliminary suggestions on which scanning behaviour should be trained. To improve performance in search tasks, people with HH could be trained to use a systematic scanning pattern that reduces the number of repetitions. To improve performance in reading tasks, people with HH could be trained to perform relatively longer saccades towards the blind hemispace, which possibly reduces the number of saccades, fixations and repetitions as well. To improve performance in mobility tasks, specifically in driving, not only long scans towards the blind hemispace could be trained, but also long scans towards the visible hemispace, which potentially results in deeper scanning into the visible hemispace. In addition, people with HH could be instructed to shift frequently between the visible and blind hemispace, and trained to distribute their scans towards relevant task information, such as cars or other moving objects. To improve walking performance, people should be instructed to scan their blind hemispace more often and create a larger dispersion of scanning by also making long scans to both hemispaces. Note that these suggestions are based on limited data on performance-enhancing scanning behaviour in search and reading tasks. Furthermore, only one walking study reported on performance-enhancing scanning behaviour. Thus, more research should first be performed on task-specific performance-enhancing scanning behaviour before implementing these suggestions for training.

### Limitations

This review has a few limitations. First, a low-quality score was found for six articles that provided information on performance-enhancing scanning behaviour in search tasks, spontaneous adaptation in search tasks, or spontaneous adaptations in reading tasks. Hence, the outcomes of those articles are less reliable. This could especially have influenced the outcomes of performance-enhancing scanning behaviour in search tasks and spontaneous adaptations in scanning behaviour in reading tasks because half of the articles had a low quality score. Nevertheless, we did not exclude these articles because the aim of this review was to provide an overview of all scanning behaviour in people with HH. Second, as previously mentioned, there is only a limited number of articles on performance-related scanning behaviour in reading and search tasks, and even less research has been done on spontaneous adaptations and training-induced alterations of scanning behaviour in mobility tasks. This limits the possibility to provide recommendations for the development of optimal rehabilitation programs for people with HH. Third, due to the lack of gaze-in-world reference systems in studies on mobility tasks it is difficult to translate findings on scanning behaviour to real-life. Fourth, definitions of fixations may have differed between articles which could have influenced the reported outcomes [20]. Finally, many different parameters have been used in previous studies to define scanning behaviour, making it difficult to compare findings on scanning behaviour between studies. As has been suggested in a previous study [17], research could benefit from using a common set of scanning parameters to assess certain aspects of scanning behaviour.

### Conclusions

In this systematic review, we summarised the literature on performance-enhancing scanning behaviour, spontaneous adaptations, and training-induced adaptations in scanning behaviour in search, reading, and mobility tasks. While we did find some similarities in performance-enhancing scanning behaviour, the outcomes suggest that this behaviour is often task-specific. Not all people with HH spontaneously develop the appropriate scanning behaviour to improve task performance. Compensatory scanning training, however, can induce scanning behaviour that enhances performance. When conducting research on scanning behaviour, it is important to report a detailed description of the parameters and task, time-dependent scanning parameters, and correlation between scanning parameters. Particularly, research should perform subgroup or single case analysis to be able to create training methods that are tailored to the individual. It is important to note that there is still a big knowledge gap on task-specific scanning behaviour in people with HH. Without a complete overview current rehabilitation programs may not yet be optimal for improving search, reading, and mobility tasks. Still, we were able to report suggestions for training scanning behaviour in people with HH from the currently available literature.

## Supplementary Material

Supplemental Material

Supplemental Material

Supplemental Material

Supplemental Material
