# Supplementary material for: A systematic review on visual scanning behaviour in hemianopia considering task specificity, performance improvement, spontaneous and training-induced adaptations
Source: Disabil Rehabil. 2023 Aug 10;46(15):3221–42. doi: 10.1080/09638288.2023.2243590 (PMC11259206; doi:10.1080/09638288.2023.2243590)
Supplement: Supplemental Material [file IDRE_A_2243590_SM5949.docx]

Definition list:

| *Scanning, task, and exercises* |  |
| --- | --- |
| Scanning | The term scanning is used in the broad sense of the word, i.e. referring to all types of viewing behaviour made during activities. |
| Task | Tasks are designed and used to experimentally investigate the performance of an activity. For example, the dot-counting task is a search tasks by which search performance is examined, reading a specific paragraph of a news article is a reading task by which reading performance is examined, and walking through an obstacle parcourse is a mobility task by which mobility performance is examined |
| Exercises | Exercises are performed within compensatory scanning training with the learning goal of applying more efficient scanning behaviours. The performed exercises could relate to daily-life activities, such as mobility, reading and searching, or an exercise could focus on employing a specific scanning behaviour, such as making longer saccades or using a systematic scanning pattern. |
| *Training methods* | |
| Visual scanning training | A training method in which people are instructed and trained to alter their scanning behaviour. |
| Visual scanning training with search exercises | A training method in which people are instructed and trained to alter their scanning behaviour during search exercises specifically. |
| Visual scanning training with reading exercises. | A training method in which people are instructed and trained to alter their scanning behaviour during reading exercises specifically. |
| Visual search training | A training method in which people are trained by performing search exercises without any instruction on how they should scan. |
| Text reading training | A training method in which people are trained by performing reading exercises without any instruction on how they should scan. |
| Audio visual stimulation training | A training method in which people are stimulated to look more into their blind visual hemispace by using both auditory and visual cues. |
| *Types of scanning behaviour we are investigating* | |
| Performance-enhancing scanning behaviour | Scanning behaviour that may improve performance in a specific task, since it was found to be related to task performance. |
| Spontaneous adaptations in scanning behaviour | Scanning behaviour that is adopted spontaneously (i.e. without training). |
| Training-induced adaptations in scanning behaviour | Training-induced adaptations are changes in scanning behaviour due to training that can both occur consciously and unconsciously. An example of such a training-induced adaptation in scanning behaviour is making longer saccades after training compared to before training. |
| *Main tasks reported in the article* | |
| Search tasks | Tasks in which participants have to find specific features or objects. |
| Reading tasks | Tasks in which participants have to read a text or several words. |
| Mobility tasks | Tasks in which subjects have to move through an environment while detecting task-relevant visual information. |
| *Scanning characteristics* | |
| Number of events | Number of scanning events, such as the number of fixations, saccades and head movements. |
| Repetitions | Repetitions of scanning, such as number of repetitions of fixations or repetitions of scanpath. |
| Duration of events | Duration of scanning events, such as fixation duration, saccadic duration |
| Length of scans | Length of scanning (events), such as total scanpath length, saccadic or head movement amplitude |
| Area distribution of scanning | How scanning behaviour is distributed in the environment, such as proportion of fixations in the blind hemispace, proportion fixations in the central area |
| Dispersion of scanning | The dispersion of scanning an environment, such as variance in fixation |
| Scanning span | The span of scans, such as mean glance depth into the blind hemispace from midline |
