# Supplementary material for: A systematic review on visual scanning behaviour in hemianopia considering task specificity, performance improvement, spontaneous and training-induced adaptations
Source: Disabil Rehabil. 2023 Aug 10;46(15):3221–42. doi: 10.1080/09638288.2023.2243590 (PMC11259206; doi:10.1080/09638288.2023.2243590)
Supplement: Supplemental Material [file IDRE_A_2243590_SM5946.docx]

# Supplementary material appendix C

Table S17. Cohen’s kappa agreement of the quality assessment

|  | Searching | | Reading | | Mobility | | Total | |
| --- | --- | --- | --- | --- | --- | --- | --- | --- |
|  | N | Cohen’s kappa | N | Cohen’s kappa | N | Cohen’s kappa | N | Cohen’s kappa |
| Performance enhancing scanning behaviour | 2 | 0.73 | 1 | 0.70 | 6 | 0.85 | 9 | 0.80 |
| Spontaneous adaptation in scanning behaviour | 7 | 0.65 | 6 | 0.55 | 3 | 0.77 | 16 | 0.64 |
| Training-induced adaptations in scanning behaviour | 6 | 0.87 | 5 | 0.80 | 0 | - | 11 | 0.83 |
| Total | 15 | 0.72 | 12 | 0.66 | 9 | 0.82 | 35 | 0.73 |
