# Supplementary material for: A systematic review on visual scanning behaviour in hemianopia considering task specificity, performance improvement, spontaneous and training-induced adaptations
Source: Disabil Rehabil. 2023 Aug 10;46(15):3221–42. doi: 10.1080/09638288.2023.2243590 (PMC11259206; doi:10.1080/09638288.2023.2243590)
Supplement: Supplemental Material [file IDRE_A_2243590_SM5943.docx]

# Supplementary material appendix B

Table S9. Quality assessment of articles reporting on performance-enhancing scanning behaviour in search.

|  | [8] | [18] |
| --- | --- | --- |
| Q1. Were the groups comparable other than the presence of disease in cases or the absence of disease in controls? | 0 | 1 |
| Q2. Were cases and controls matched appropriately? | 0 | 1 |
| Q3. Were the same criteria used for identification of cases and controls? | ? | ? |
| Q4. Was exposure measured in a standard, valid and reliable way? | 1 | 1 |
| Q5. Was exposure measured in the same way for cases and controls? | 1 | 1 |
| Q6. Were confounding factors identified? | 0 | 0 |
| Q7. Were strategies to deal with confounding factors stated? | NA | NA |
| Q8. Were outcomes assessed in a standard, valid and reliable way for cases and controls? | 1 | 1 |
| Q9. Was the exposure period of interest long enough to be meaningful? | 0 | 1 |
| Q10. Was appropriate statistical analysis used? | 1 | 1 |
| Total score | 4 | 7 |

1=yes, 0=no, ?=don’t know, NA=not applicable. Total score is the sum of all 1’s.

Table S10. Quality assessment of articles reporting on spontaneous adaptations in scanning behaviour in search.

|  | [2] | [23] | [30] | [28] | [31] | [27] | [38] |
| --- | --- | --- | --- | --- | --- | --- | --- |
| Q1. Were the groups comparable other than the presence of disease in cases or the absence of disease in controls? | 0 | 1 | 1 | 0 | ? | 1 | 0 |
| Q2. Were cases and controls matched appropriately? | 0 | 0 | 1 | 0 | 0 | 0 | 0 |
| Q3. Were the same criteria used for identification of cases and controls? | 1 | 1 | ? | 1 | 1 | 1 | 1 |
| Q4. Was exposure measured in a standard, valid and reliable way? | 1 | 1 | 1 | 0 | 1 | 1 | 1 |
| Q5. Was exposure measured in the same way for cases and controls? | 1 | 1 | 1 | 1 | 1 | 1 | 1 |
| Q6. Were confounding factors identified? | 1 | 0 | 0 | 0 | 0 | 0 | 0 |
| Q7. Were strategies to deal with confounding factors stated? | 1 | NA | NA | NA | NA | NA | NA |
| Q8. Were outcomes assessed in a standard, valid and reliable way for cases and controls? | 1 | 1 | 1 | 1 | 1 | 1 | 1 |
| Q9. Was the exposure period of interest long enough to be meaningful? | 1 | 1 | 1 | 0 | 1 | 1 | 1 |
| Q10. Was appropriate statistical analysis used? | 1 | ? | 1 | 1 | 1 | 1 | 1 |
| Total score | 8 | 6 | 7 | 4 | 6 | 7 | 6 |

1=yes, 0=no, ?=don’t know, NA=not applicable. Total score is the sum of all 1’s.

Table S11. Quality assessment of articles reporting on training-induced adaptations in scanning behaviour in search.

|  | [8] | [13] | [25] | [29] | [34] | [28] |
| --- | --- | --- | --- | --- | --- | --- |
| Q1. Is it clear in the study what is the ‘cause’ and what is the ‘effect’ (i.e. there is no confusion about which variable comes first)? | 1 | 1 | 1 | 1 | 1 | 1 |
| Q2. Were the participants included in any comparisons similar? | 1 | 1 | 1 | 1 | 1 | 1 |
| Q3. Were the participants included in any comparisons receiving similar treatment/care, other than the exposure or intervention of interest? | 1 | 1 | 1 | 1 | 1 | 1 |
| Q4. Was there a control group? | 0 | 1 | 0 | 0 | 0 | 0 |
| Q5. Were there multiple measurements of the outcome both pre and post the intervention/exposure? | 0 | 0 | 0 | 0 | 1 | 0 |
| Q6. Was follow up complete and if not, were differences between groups in terms of their follow up adequately described and analyzed? | NA | 0 | 1 | 0 | 1 | NA |
| Q7. Were the outcomes of participants included in any comparisons measured in the same way? | 1 | 1 | 1 | 1 | 1 | 1 |
| Q8. Were outcomes measured in a reliable way? | 1 | 1 | 1 | 1 | 1 | 1 |
| Q9. Was appropriate statistical analysis used? | 1 | 1 | 1 | 1 | 1 | 1 |
| Total score | 6 | 7 | 7 | 6 | 8 | 6 |

1=yes, 0=no, ?=don’t know, NA=not applicable. Total score is the sum of all 1’s.

Table S12. Quality assessment of articles reporting on performance-enhancing scanning behaviour in reading.

|  | [40] |
| --- | --- |
| Q1. Were the groups comparable other than the presence of disease in cases or the absence of disease in controls? | 1 |
| Q2. Were cases and controls matched appropriately? | 1 |
| Q3. Were the same criteria used for identification of cases and controls? | 1 |
| Q4. Was exposure measured in a standard, valid and reliable way? | 1 |
| Q5. Was exposure measured in the same way for cases and controls? | 1 |
| Q6. Were confounding factors identified? | 0 |
| Q7. Were strategies to deal with confounding factors stated? | NA |
| Q8. Were outcomes assessed in a standard, valid and reliable way for cases and controls? | 1 |
| Q9. Was the exposure period of interest long enough to be meaningful? | 1 |
| Q10. Was appropriate statistical analysis used? | 1 |
| Total score | 8 |

1=yes, 0=no, ?=don’t know, NA=not applicable. Total score is the sum of all 1’s.

Table S13. Quality assessment of articles reporting on spontaneous adaptations in scanning behaviour in reading

|  | [24] | [31] | [36] | [37] | [38] | [40] |
| --- | --- | --- | --- | --- | --- | --- |
| Q1. Were the groups comparable other than the presence of disease in cases or the absence of disease in controls? | 1 | ? | 0 | 1 | 1 | 1 |
| Q2. Were cases and controls matched appropriately? | 0 | 0 | 0 | 0 | 1 | 1 |
| Q3. Were the same criteria used for identification of cases and controls? | 1 | 1 | 1 | ? | 1 | 1 |
| Q4. Was exposure measured in a standard, valid and reliable way? | 1 | 0 | 1 | 0 | 0 | 0 |
| Q5. Was exposure measured in the same way for cases and controls? | 1 | 1 | 1 | 1 | 1 | 1 |
| Q6. Were confounding factors identified? | 0 | 0 | 0 | 0 | 0 | 0 |
| Q7. Were strategies to deal with confounding factors stated? | NA | NA | NA | NA | NA | NA |
| Q8. Were outcomes assessed in a standard, valid and reliable way for cases and controls? | 1 | 1 | 1 | 1 | 1 | 1 |
| Q9. Was the exposure period of interest long enough to be meaningful? | 1 | 1 | 0 | 1 | 1 | 1 |
| Q10. Was appropriate statistical analysis used? | 1 | 1 | 1 | ? | 1 | 1 |
| Total score | 7 | 5 | 5 | 4 | 7 | 7 |

1=yes, 0=no, ?=don’t know, NA=not applicable. Total score is the sum of all 1’s.

Table S14. Quality assessment of articles reporting on training-induced adaptations in scanning behaviour in reading

|  | [13] | [29] | [31] | [39] | [40] |
| --- | --- | --- | --- | --- | --- |
| Q1. Is it clear in the study what is the ‘cause’ and what is the ‘effect’ (i.e. there is no confusion about which variable comes first)? | 1 | 1 | 1 | 1 | 1 |
| Q2. Were the participants included in any comparisons similar? | 1 | 1 | 1 | 0 | 1 |
| Q3. Were the participants included in any comparisons receiving similar treatment/care, other than the exposure or intervention of interest? | 1 | 1 | 1 | 1 | 1 |
| Q4. Was there a control group? | 1 | 0 | 1 | 1 | 0 |
| Q5. Were there multiple measurements of the outcome both pre and post the intervention/exposure? | 0 | 0 | 0 | 0 | 0 |
| Q6. Was follow up complete and if not, were differences between groups in terms of their follow up adequately described and analysed? | 0 | NA | 1 | 1 | NA |
| Q7. Were the outcomes of participants included in any comparisons measured in the same way? | 1 | 1 | 1 | 1 | 1 |
| Q8. Were outcomes measured in a reliable way? | 1 | 1 | 1 | 1 | 1 |
| Q9. Was appropriate statistical analysis used? | 1 | 1 | 1 | 1 | 1 |
| Total score | 7 | 6 | 8 | 7 | 6 |

1=yes, 0=no, ?=don’t know, NA=not applicable. Total score is the sum of all 1’s.

Table S15. Quality assessment of articles reporting on performance-enhancing scanning behaviour in mobility.

|  | [57] | [49] | [55] | [54] | [56] | [53] |
| --- | --- | --- | --- | --- | --- | --- |
| Q1. Were the groups comparable other than the presence of disease in cases or the absence of disease in controls? | 1 | 1 | 1 | 1 | 1 | 1 |
| Q2. Were cases and controls matched appropriately? | 1 | 1 | 1 | 1 | 1 | 1 |
| Q3. Were the same criteria used for identification of cases and controls? | 1 | 1 | 1 | 1 | 1 | 1 |
| Q4. Was exposure measured in a standard, valid and reliable way? | 1 | 1 | 1 | 1 | 0 | 1 |
| Q5. Was exposure measured in the same way for cases and controls? | 1 | 1 | 1 | 1 | 1 | 1 |
| Q6. Were confounding factors identified? | 0 | 0 | 0 | 0 | 0 | 0 |
| Q7. Were strategies to deal with confounding factors stated? | NA | NA | NA | NA | NA | NA |
| Q8. Were outcomes assessed in a standard, valid and reliable way for cases and controls? | 1 | 1 | 0 | 1 | 0 | 1 |
| Q9. Was the exposure period of interest long enough to be meaningful? | 1 | 1 | 0 | 1 | 1 | 1 |
| Q10. Was appropriate statistical analysis used? | 1 | 1 | 1 | 1 | 1 | 1 |
| Total score | 8 | 8 | 6 | 8 | 6 | 8 |

1=yes, 0=no, ?=don’t know, NA=not applicable. Total score is the sum of all 1’s.

Table S16. Quality assessment of articles reporting on spontaneous adaptations in scanning behaviour in mobility.

|  | [27] | [48] | [52] |
| --- | --- | --- | --- |
| Q1. Were the groups comparable other than the presence of disease in cases or the absence of disease in controls? | 1 | 1 | 1 |
| Q2. Were cases and controls matched appropriately? | 0 | 1 | 1 |
| Q3. Were the same criteria used for identification of cases and controls? | 1 | ? | 1 |
| Q4. Was exposure measured in a standard, valid and reliable way? | 1 | 1 | 1 |
| Q5. Was exposure measured in the same way for cases and controls? | 1 | 1 | 1 |
| Q6. Were confounding factors identified? | 0 | 0 | 0 |
| Q7. Were strategies to deal with confounding factors stated? | NA | NA | NA |
| Q8. Were outcomes assessed in a standard, valid and reliable way for cases and controls? | 1 | 1 | 1 |
| Q9. Was the exposure period of interest long enough to be meaningful? | 1 | 1 | 1 |
| Q10. Was appropriate statistical analysis used? | 1 | 1 | 1 |
| Total score | 7 | 7 | 8 |

1=yes, 0=no, ?=don’t know, NA=not applicable. Total score is the sum of all 1’s.
