# Supplementary material for: A systematic review on visual scanning behaviour in hemianopia considering task specificity, performance improvement, spontaneous and training-induced adaptations
Source: Disabil Rehabil. 2023 Aug 10;46(15):3221–42. doi: 10.1080/09638288.2023.2243590 (PMC11259206; doi:10.1080/09638288.2023.2243590)
Supplement: Supplemental Material [file IDRE_A_2243590_SM5937.docx]

# Supplementary material appendix A

Table S1. Descriptives of articles reporting spontaneous adaptations in scanning behaviour during imagery tasks.

| Article | N participant | Gender (%M) | Age (M±SD) | right hemifield | Left hemifeld | Quadrantanopia | Duration of VFD | Task |
| --- | --- | --- | --- | --- | --- | --- | --- | --- |
| [60] | HHR=3 | 66 | 44-67^c^ | 3 | 0 | 0 | 30-78 months^c^ | The visual imagery task: The participants were asked to imagine a map of France. They had to name as many French towns as they could visualize on their imagined map beginning with Paris. The visually guided task: The participants were asked to look at a geographical map of France and to name towns that they were able to locate on the map beginning with Paris. |
|  | HHL=2 | 100 | 60-71^c^ | 0 | 1 | LT=1 | 1-138 months^c^ |  |
|  | UV=10 | 50 | nm |  |  |  |  |  |
| [64,65] | HH=10 |  | 52±7 |  |  |  | >3 months | Participants were asked to look carefully at pictures and recall their specific features. Afterwards the participants had to describe the picture content and provide some details on request |
|  | UV=10 |  | 35±6 |  |  |  |  |  |

nm = not mentioned, HH= homonymous hemianopia, UV = unimpaired vision.

1. RT= right top, RB = right bottom, LT = left top, LB = left bottom
2. Mean (range)
3. Range
4. Mean

Table S2. Spontaneous adaptations in scanning behaviour in imagery tasks.

|  | Reference | [60] | [64,65] |
| --- | --- | --- | --- |
|  | Reference system | EiH | GiW |
|  | head restrained | Yes | Yes |
| Number of events | Number of saccades total | n.s.^a^ | - |
| Scanning span | Bias towards blind hemifield | *HH>UV |  |
| Dispersion of scanning | Global/local ratio saccades |  | ***HH<UV |

* p<0.05, ** P<0.01, ***P<0.001, + trend p<0.1, n.s. not significant, - no results reported, EiH eye-in-head, GiW gaze-in-world, HH = Homonymous hemianopia, UV = people with unimpaired vision.

a. No difference in the number of saccades towards the blind hemispace as well.

Table S3. Descriptives of articles reporting spontaneous adaptations in scanning behaviour during natural viewing tasks.

| Article | N participant | Gender (%M) | Age (M±SD) | right hemifield | Left hemifeld | Quadrant-  anopia^a^ | Duration of VFD | | Task | |  |
| --- | --- | --- | --- | --- | --- | --- | --- | --- | --- | --- | --- |
| [67] | HH=8 | 50 | 30-76 | 0 | 2 | LT=1, LB=5 | | 46 - 1599 days^c^ | | Participants were asked to view a virtual traffic scene, displaying an intersection environment, with a two-way road and a pedestrian-crossing surrounded by buildings and trees. The virtual scene was presented in a static and dynamic condition | |
|  | UV=8 | 63 | 25-78 |  |  |  |  | |  | |  |
| [68] | HH=10 | 50 | 49±17 | 0 | 10 | 0 | 165±251days | | Each participant viewed thirty images that were selected randomly either among the original images or their mirrored versions. | |  |
|  | UV=10 | 40 | 61±8 |  |  |  |  | |  |  |  |
| [71] | HH=8 | nm | 34.5 (24-51) | 4 | 4 | 0 | nm | | The participants were asked to view 44 filtered or unfiltered images that were displayed for 3s each. | |  |
|  | UV=8 | nm | 36.9 (24-48) | |  |  |  | |  |  |  |

nm = not mentioned, HH= homonymous hemianopia, HHL= homonymous hemianopia left, HHR= homonymous hemianopia right, UV = unimpaired vision, RT= right top, RB = right bottom, LT = left top, LB = left bottom

1. Mean (range)
2. Range
3. Mean

Table S4. Spontaneous adaptations in scanning behaviour in natural viewing tasks.

|  | Reference | [67] | [68] | [71] |
| --- | --- | --- | --- | --- |
|  | Reference system | GiH | GiH | GiH |
|  | Head fixed | No | - | Yes |
| Number of events | Number of fixations total | - | - | *** HH>UV^a^ |
| Repetitions | Percentage of item refixations total | - | - | n.s. |
| Duration of events | Fixation duration total | - | - | ***HH<UV^a^ |
|  | Fixation duration first fixation | - | - | n.s. |
| Length of scans | Scan path length | - | - | ***HH>UV |
|  | Mean saccadic amplitude | - | - | n.s. |
|  | Mean saccadic amplitude first saccade | - | - | ***HH>UV |
|  | Mean saccadic amplitude hemi-space visible | n.s. | - | - |
| Area distribution of scanning | Number of fixations in blind hemifield | - | - | *HH>UV^a^ |
|  | Fixation duration in hemispace blind | n.s.^b^ | *HH>UV | *HH>UV |
|  | Number of saccades directed towards the blind hemifield | n.s. | - | *HH>UV |
| Scanning span | Mean deviation saccades from the midline | - | - | ***HH<UV |
| Dispersion of scanning | Global / local ratio saccades | - | - | n.s. |
|  | Area of image covered by fixations | - | - | ***HH>UV |
|  | Fixation pattern similarity | - | - | *HH ~ UV |

* p<0.05, ** P<0.01, ***P<0.001, + trend p<0.1, n.s. not significant, ~not similar, - no results reported, EiH eye-in-head, GiW gaze-in-world, HH = Homonymous hemianopia, UV = people with unimpaired vision.

a. Only for filtered images

b. Fixation duration in visible hemispace also not significant

Table S5. Descriptives of articles reporting spontaneous adaptations in scanning behaviour during line bisection tasks.

| Article | N participant | | Gender (%M) | Age (M±SD) | right hemifield | Left hemifeld | Quadrantanopia | paracentral | Duration of VFD | Task |
| --- | --- | --- | --- | --- | --- | --- | --- | --- | --- | --- |
| [72] | | HHR=2 | 0 | 44 (6) | 2 | 0 | 0 | 0 | 8-18 months | Each subject was asked to examine each line entirely and then use the pointer to touch the centr of the line. Two different horizontal lines (45 and 34 degrees) were presented |
|  |  | HHL=5 | 80 | 49 (19) | 0 | 5 | 0 | 0 | 0.25 - 90 monts |  |
|  |  | UV=9 | nm | 59(3) |  |  |  |  |  |  |
| [73] | | HHR=8 | 88 | 60 (10) | 8 | 0 | 0 | 0 | nm | Each subject performed the line bisection task. The lines were 20cm or 15 cm long, and placed in the centre, right hemispace or left hemispace. |
|  |  | HHL=5 | 60 | 59(7) | 0 | 5 | 0 | 0 | nm |  |
|  |  | UV=10 | nm | 61(10) |  |  |  |  |  |  |

nm = not mentioned, HH= homonymous hemianopia, HHL= homonymous hemianopia left, HHR= homonymous hemianopia right, UV = unimpaired vision

1. RT= right top, RB = right bottom, LT = left top, LB = left bottom
2. Mean (range)
3. Range
4. Mean

Table S6. Spontaneous adaptations in scanning behaviour in line bisection tasks.

|  | Reference | [72] | [73] |  |
| --- | --- | --- | --- | --- |
|  | Reference system | GiW | GiW | |
|  | Head fixed | Yes | No | |
| Number of events | Number of fixations | n.s. | - | |
|  | Number of saccades | n.s. | - | |
| Length of scans | Mean saccadic amplitude | *HH>UV | - | |
| Are distribution of scanning | Percentage of searches in the blind hemispace | - | **HH>UV | |
| Scanning span | Fixation location towards blind hemifield | **HH>UV^a^ | - | |
|  | Fixation range | n.s. | - | |

* p<0.05, ** P<0.01, ***P<0.001, + trend p<0.1, n.s. not significant, - no results reported, EiH eye-in-head, GiW gaze-in-world, HH = Homonymous hemianopia, UV = people with unimpaired vision

a. For midpoint fixation locations all fixations and fixation with the longest duration, furthest fixation location, median fixation location.

Table S7. Descriptives of articles reporting spontaneous adaptations in scanning behaviour during creating tasks.

| Article | N participant | Gender (%M) | Age (M±SD) | right hemifield | Left hemifeld | Quadrantanopia | paracentral | Duration of VFD | Task |
| --- | --- | --- | --- | --- | --- | --- | --- | --- | --- |
| [76] | HH=3 | 66^d^ | 50-84^c^ | 2 | 1 | 0 | 0 | 8-24 months^c^ | The participants were asked to build a standardized model |
|  | UV=4 | 50^d^ | 50-55^c^ |  |  |  |  |  |  |

nm = not mentioned, HH= homonymous hemianopia, UV = unimpaired vision

1. RT= right top, RB = right bottom, LT = left top, LB = left bottom
2. Mean (range)
3. Range
4. Mean

Table S8. Spontaneous adaptations in scanning behaviour in creating tasks.

|  | reference | [76] |
| --- | --- | --- |
|  | Reference system | EiH |
|  | Head fixed | No |
| Number of events | Number of fixations on distractor locations | *HH>UV |
|  | look ahead fixations | n.s. |
|  | look back fixations | n.s. |
| Duration of events | Fixation duration total | n.s. |
|  | Saccadic duration | n.s. |
|  | Saccadic peak velocity | n.s. |
| Length of scans | Mean saccadic amplitude | n.s. |

* p<0.05, ** P<0.01, ***P<0.001, + trend p<0.1, n.s. not significant, - no results reported, EiH eye-in-head, GiW gaze-in-world, HH = Homonymous hemianopia, UV = people with unimpaired vision.
